# Application of Covalent Organic Frameworks in Sulfur‐Based Battery Separators

**DOI:** 10.1002/smsc.202300056

**Published:** 2023-08-13

**Authors:** Yuluan Zhang, Can Guo, Luanhua Zhou, Xiaoman Yao, Yiwen Yang, Huifen Zhuang, Yi-Rong Wang, Qing Huang, Yifa Chen, Shun-Li Li, Ya-Qian Lan

**Affiliations:** ^1^ School of Chemistry South China Normal University Guangzhou 510006 P. R. China; ^2^ College of Materials Science and Engineering Nanjing Forestry University Nanjing 210037 P. R. China

**Keywords:** covalent organic frameworks, modification, separators, sulfur-based batteries

## Abstract

Owing to the high storage capacity and multiple‐electron‐transfer chemistry of sulfur (S), sulfur‐based (S‐based) batteries with merits of high theoretical capacity/energy density, eco‐friendliness, and abundant supply hold much potential for energy‐storage/conversion devices. The capacity of S‐based batteries is much higher than that of traditional‐metal‐oxide cathode‐based lithium‐ion batteries, which is regarded as the highest capacity of solid‐state cathode materials at the current stage. As a vital component of S‐based batteries, separators play a profound role in resolving urgent issues (e.g., shuttling effect, volume expansion, poor conductivity, and metal dendrites, etc.). So far, some pioneering works have been reported in the exploration of separators for S‐based battery. On this basis, covalent organic frameworks (COFs), as a kind of functional materials, offer much possibility for S‐based battery separators due to their advantages such as high porosity, crystalline and well‐defined structures, designable struts, and tunable functions, etc. Herein, the reported works about COFs in S‐based battery separators including their structural characteristics, preparation methods, application forms, and battery properties are summarized. Moreover, also, a brief perspective is proposed on the challenges of COFs applied in S‐based battery separators, and new insights are provided for scientists in this field.

## Graphical abstract

The reported work on applications of covalent organic frameworks (COFs) in sulfur‐based battery separators is summarized and their structural characteristics, preparation methods, application forms, and battery properties are discussed.

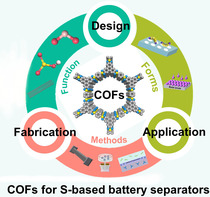

## 1 Introduction

Sulfur (S), as a by‐product of the petroleum industry, can serve as one of the most prospective electrode materials for next‐generation energy‐storage devices on account of its attractive properties of low cost, environmental benign, abundant supply, and high theoretical capacity.^[^ ^1^, ^2^ ^]^ Specifically, a capacity of 1675 mAh g^−1^ could be delivered owing to the reduction reaction of S, which is larger than typical insertion‐type cathode materials.^[^ ^3^ ^]^ Especially when S cathodes are paired with lithium (Li) anodes, the obtained lithium–sulfur (Li–S) battery presents theoretical energy densities of 2600 Wh kg^−1^,^[^ ^4^, ^5^ ^]^ higher than lithium‐ion batteries (LIBs) (LiCoO_2_/graphite batteries, 387 Wh kg^−1^), or its derivative battery systems (e.g., Li‐CO_2_ battery, 1876 Wh kg^−1^).^[^ ^6^, ^7^, ^8^, ^9^ ^]^ Thus, during the past few decades, S has been applied as promising cathode material to be cooperated with diverse anodes, such as multivalent metals like Ca, Mg, and Al or alkali metals like Li, Na, and K. Owing to these advantages, S‐based battery holds much promise in a number of potential applications (e.g., electric vehicles and unmanned aircraft systems, etc.).^[^ ^10^, ^11^ ^]^ Specifically, Li–S batteries with 350 Wh kg^−1^ have been explored and tested in small‐scale commercial applications by OXIS Energy and Sion Power.^[^ ^12^ ^]^ In spite of these progresses in S‐based battery, it still faces obstacles in mass production, which is ascribed to the inherent drawbacks of S‐based battery (e.g., slow reaction kinetics, poor conductivity, shuttling effect of polysulfides (PSs), metal dendrites, etc.).^[^ ^13^, ^14^, ^15^ ^]^

In general, the multi‐step conversion of sulfur gives a high specific capacity to S‐based battery yet it is also an inherent drawback that limits the application of S‐based battery. Taking the Li–S battery as an example, the discharging process contains the conversion of S_8_ to Li_2_S, during which it can be mainly divided into the stepwise reduction of S_8_ to long‐chain PSs and further into low‐order PSs, resulting in specific capacity of 419 and 1256 mAh g^−1^ respectively. All of these processes give an overall theoretical specific capacity of 1675 mAh g^−1^. However, the simultaneously generated shuttling PSs, rapid consumption of active materials and the dendrite behaviors largely affect the battery performance and remain to be inherent bottlenecks that largely restrict the potential applications of Li–S batteries.

To conquer these bottlenecks, diverse strategies have been explored to promote the properties of S‐based batteries including the development of innovative S host materials, separator modification, binder optimization, and solid‐state electrolytes, etc.^[^ ^16^, ^17^, ^18^, ^19^, ^20^, ^21^, ^22^ ^]^ Among them, the separator, as the core component of the S‐based battery, possesses vital functions of cathode and anode separation, short circuit prevention, and metal‐ion diffusion, whose performances are strongly related to the active material utilization rate, internal resistance, safety, and battery performances.^[^ ^23^ ^]^ An ideal separator needs to meet the requirements like chemical stability, thermal stability and sufficient mechanical strength, meanwhile it can play a protective role in microporous self‐closing under high temperatures.^[^ ^24^ ^]^ In addition to these, the functions like the PSs adsorption/catalysis or anode protection are also essential for the separators that can be applied in S‐based battery.^[^ ^25^ ^]^ Battery separators can be generally classified into three types based on their structure and composition: microporous polymer membranes, non‐woven membranes, and inorganic composite membranes.^[^ ^26^ ^]^ Among them, due to their advantages in cost, performance and safety, microporous polymer membranes are most widely investigated separators in liquid electrolyte battery.^[^ ^27^ ^]^ The type of microporous polymer separator can be divided into polyolefin, poly(ethylene oxide) (PEO),^[^ ^28^, ^29^ ^]^ polyvinylidene fluoride (PVDF),^[^ ^30^, ^31^ ^]^ poly(acrylonitrile) (PAN)^[^ ^32^, ^33^ ^]^ and co‐blended polymer based separators, etc.^[^ ^19^, ^20^ ^]^ During past years, traditional polymers like PEO or PVDF have been studied as separators in Li battery systems, yet they are still limited by their inherent drawbacks. For example, the regularity and crystallization tendency of PEO separators can hinder Li^+^ migration and lead to low conductivity, while the inferior tensile strength and thermal stability still limit the applications of PVDF separators.^[^ ^34^, ^35^ ^]^ Compared to the above materials, polyolefin separators including polyethylene (PE) and polypropylene (PP) with mature production processes have been widely used in battery as commercialized separators with excellent chemical stability, high porosity, and low cost, etc.^[^ ^36^, ^37^, ^38^ ^]^ However, for polyolefin separators, there are multi‐requirements for their applications in S‐based battery (e.g., metal‐ion/electrolyte diffusion, inhibition of PSs shuttling, metal anode protection, or electrolyte wetting, etc.), which would not be satisfied by the merely using of single‐function polyolefin. Thus, the modification of polyolefin separators with certain functional materials becomes one of the ideal options that are effective and capable of meeting large‐scale manufacturing needs, which has arisen to be a hot topic in the battery field.^[^ ^19^, ^39^, ^40^ ^]^

Much progress has been made in the last decades on the modification of conventional separators and most of them applied function materials like carbon, inorganic or polymeric materials.^[^ ^23^, ^41^ ^]^ To achieve high‐performance separators without sacrificing the battery energy density, alternative materials with properties like lightweight or ease in processing are more desired to modify the separator. Covalent organic frameworks (COFs), a kind of functional material that composed of light organic components joined by covalent bonds, has gained significant interest globally.^[^ ^42^ ^]^ Since Omar Yaghi et al. first reported COFs in 2005, research on COFs has developed rapidly and possessed much potential in the fields of photo‐/electro‐catalysis,^[^ ^43^, ^44^, ^45^, ^46^ ^]^ gas adsorption/separation,^[^ ^47^ ^]^ sensing,^[^ ^48^ ^]^ and drug delivery systems, etc.^[^ ^49^ ^]^ In the research field of energy storage, COFs offer much possibility for the potential application in S‐based battery owing to their modifiable and well‐defined structures with high porosity that are much beneficial for the rapid transfer of metal ions/electrolyte and the design of functional units to promote the battery performance.^[^ ^50^, ^51^, ^52^, ^53^, ^54^ ^]^ However, there are still some bottlenecks: 1) poor conductivity that might increase the impedance and affect the charge transfer; 2) the interaction between soluble PSs and COFs is weak and requires the specific design and exploitation of effective functional groups; and 3) most of COFs are synthesized by solvothermal reactions in hermetically sealed glass tubes at certain temperatures, which is still limited in scalable production or cost. Therefore, strategies that can address these issues and explore the potential applications of COFs in the modification of separators are highly demanded.

**Figure 1:**
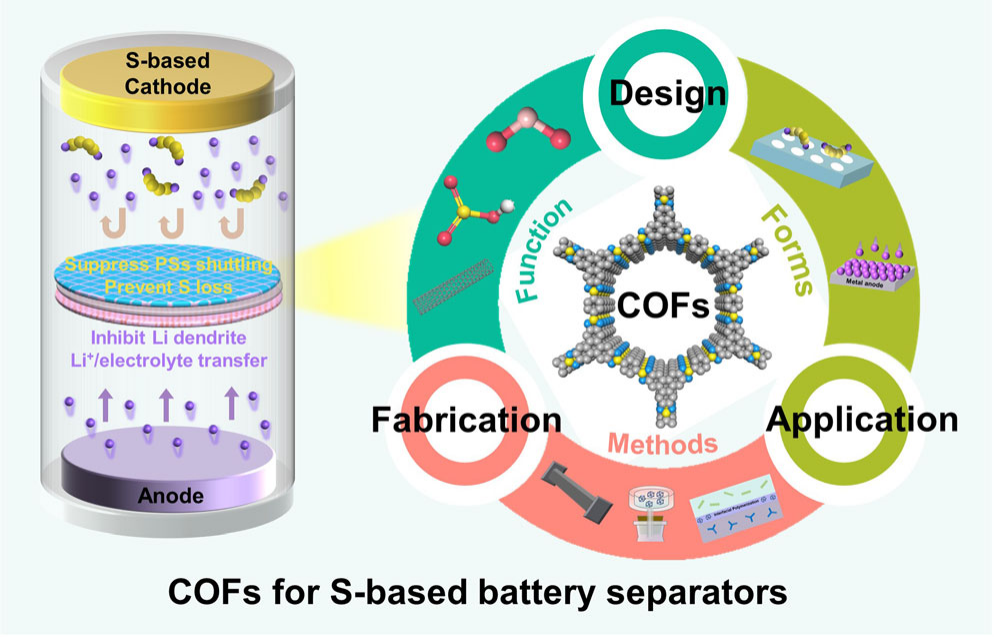
Schematic representation of covalent organic frameworks (COFs) for S‐based battery separators.

This work will systematically review the recent progress of advanced COFs‐modified S‐based battery separators, including the summary of the structure design of COFs, preparation methods, application forms of separators, and the relationship between COFs structures and electrochemical performances, etc. (**Figure** **1**). In the first part, this review first comments the development history and structural characteristics of COFs for S‐based battery separators. Then, it summarizes the preparation methods of COFs‐modified separators containing casting, vacuum filtration, and interfacial in situ polymerization. After that, it presents a systematic review on the design and performance of S‐based battery systems, including Li–S, Na–S, and Li–SeS_2_ battery, based on the requirements of anodic and cathodic electrodes. Finally, the prospects and possible research directions in this field are also discussed.

## 2 Application of COFs in S‐Based Battery Separators

### 2.1 The Types and Timeline of COFs for S‐Based Battery Separators

COFs are typically constructed from tunable structural blocks by reversible condensation reactions that allow for the precise design of functionalities for the requirements of separator modification.^[^ ^42^ ^]^ Since 2005, Yaghi first designed and successfully realized 2D COFs using the dehydration condensation of boronic acid and hexahydroxytriphenylene, a series of reversible reactions (e.g., boron chemistry, Schiff base chemistry, and triazine chemistry) for the design of COFs have been reported.^[^ ^55^, ^56^ ^]^ Among them, the Schiff base reaction, serving as the most commonly applied reaction in COF synthesis, includes the connection of ligands through imine bond, amide bond, etc. During the reaction process, various functional groups can be introduced into the COFs skeleton through specially selected of the structure struts with targeted functional groups. The common functional groups in COFs include electron‐rich atoms such as N and O, active bonds (e.g., imine [–C = N–], carbonyl [–C = O], etc.) and anionic groups such as sulfonic acid (–SO_3_H–), as well as conjugated structures (e.g., porphyrins) as shown in **Table** **1**. In S‐based battery separators, COFs are likewise involved in metal‐ion/electrolyte transport and PSs adsorption/catalysis mainly through these active functional groups. These functional groups might promote Li^+^/electrolyte transport and inhibit PSs shuttling. Some representative work examples will be given in the following section.

**Table 1:** A summary of different COFs and their functional groups for sulfur‐based battery separator modification

	Ligand 1	Ligand 2	Functional groups	Battery system	Reference
1	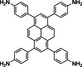	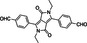	–C=N electron‐rich N/O	Li–S	[32]
2	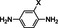 X = –H,–Cl,–SO_3_H	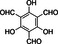	–C=N –X –OH	Li–S	[60]
3	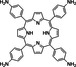	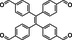	–C=N porphyrin	Li–S	[62]
4	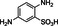	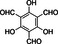	–C=N –OH –SO_3_H	Li–S	[61]
5	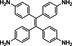	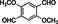	–C=N –OCH_3_	Li–S	[65]
6	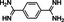	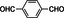	–C=N electron‐rich N	Li–S	[68]
7	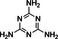	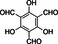	–C=N triazine	Li–S	[69]
8	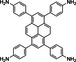	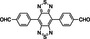	–C=N electron‐rich N/S	Li–S	[70]
9	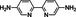	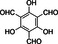	–C=N electron‐rich N	Li–S	[58]
10	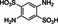	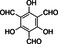	–C=N –OH –SO_3_H	Li–S	[59]
11	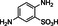	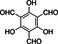	–OH –SO_3_Li	Li–S	[71, 72]
12	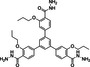	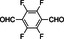	–F –C=N	Li–S	[73]
13	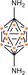	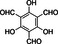	Carborane –C=O –OH	Li–S	[74]
14	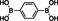	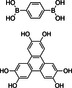	boroxine	Li–S	[57]
15	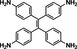	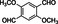	–C=N –OCH_3_	Li–S	[75]
16	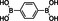	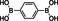	Boroxine	Li–S	[76]
17	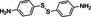	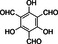	–C=N –S–S– OH	Li–S	[77]
18	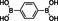	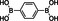	Boroxine	Li–S	[78]
19	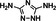	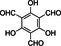	–C=N –OH electron‐rich N	Li–S	[79]
20	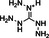	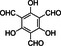	–OH electron‐rich N	Li–S	[80]
21	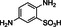	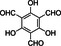	–C=N –OH –SO_3_H	Li–S	[81]
22	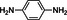	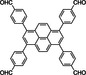	–C=N	Li–S	[82]
23	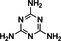	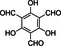	–C=N triazine	Li–S	[83]
24	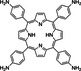	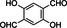	–C=N Porphyrin –OH	Li–S	[63]
25	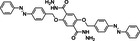	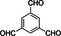	–N=N	Na–S	[88]
26	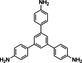	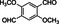	–C=N –OCH_3_	Li–SeS_2_	[92]
27	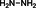	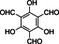	–C=N –OH	Li–SeS_2_	[93]
28	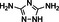	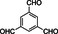	–C=N electron‐rich N	Li–SeS_2_	[94]

**Figure 2:**
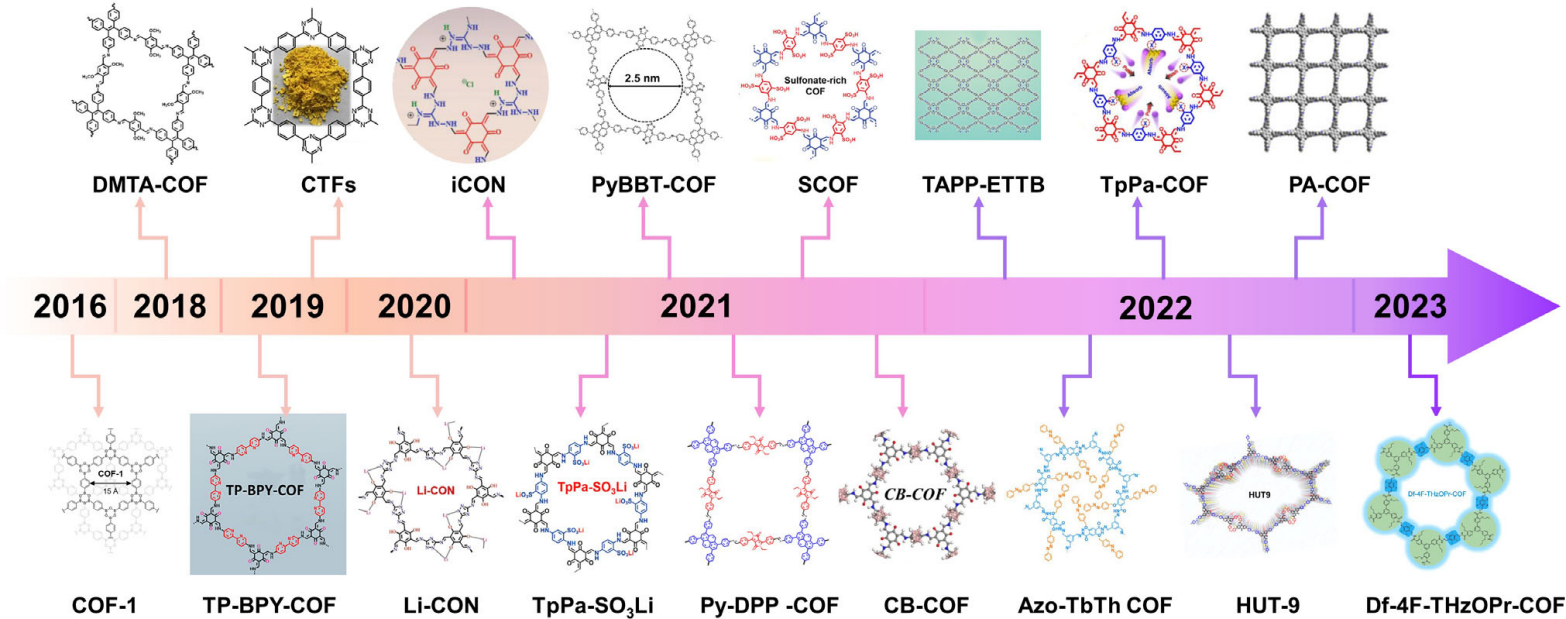
Evolution timeline of applications of COFs in S‐based battery separators. Image for Py–DPP‐COF: Reproduced with permission.^[^ ^32^ ^]^ Copyright 2021, American Chemical Society. Image for COF‐1: Reproduced with permission.^[^ ^57^ ^]^ Copyright 2016, American Chemical Society. Image for TP–Bpy‐COF: Reproduced with permission.^[^ ^58^ ^]^ Copyright 2019, American Chemical Society. Image for TpPa‐COF: Reproduced with permission.^[^ ^60^ ^]^ Copyright 2022, American Chemical Society. Image for TAPP–ETTB: Reproduced with permission.^[^ ^62^ ^]^ Copyright 2022, American Chemical Society. Image for DMTA‐COF: Reproduced with permission.^[^ ^65^ ^]^ Copyright 2018, American Chemical Society. Image for PyBBT‐COF: Reproduced with permission.^[^ ^70^ ^]^ Copyright 2021, American Chemical Society. Image for TpPa–SO_3_Li: Reproduced with permission.^[^ ^71^ ^]^ Copyright 2021, American Chemical Society. Image for Df‐4F‐THzOPr‐COF: Reproduced with permission.^[^ ^73^ ^]^ Copyright 2023, American Chemical Society. Image for CB‐COF: Reproduced with permission.^[^ ^74^ ^]^ Copyright 2021, American Chemical Society. Image for HUT‐9: Reproduced with permission.^[^ ^77^ ^]^ Copyright 2022, American Chemical Society. Image for PA‐COF: Reproduced with permission.^[^ ^82^ ^]^ Copyright 2022, American Chemical Society. Image for Azo‐TbTh COF: Reproduced with permission.^[^ ^88^ ^]^ Copyright 2022, American Chemical Society. Image for SCOF: Reproduced with permission.^[^ ^59^ ^]^ Copyright 2021, Wiley‐VCH. Image for iCONs: Reproduced with permission.^[^ ^80^ ^]^ Copyright 2021, Wiley‐VCH. Image for CTFs: Reproduced with permission.^[^ ^68^ ^]^ Copyright 2019, Elsevier. Image for Li‐CONs: Reproduced with permission.^[^ ^79^ ^]^ Copyright 2020, Elsevier.

**Figure 3:**
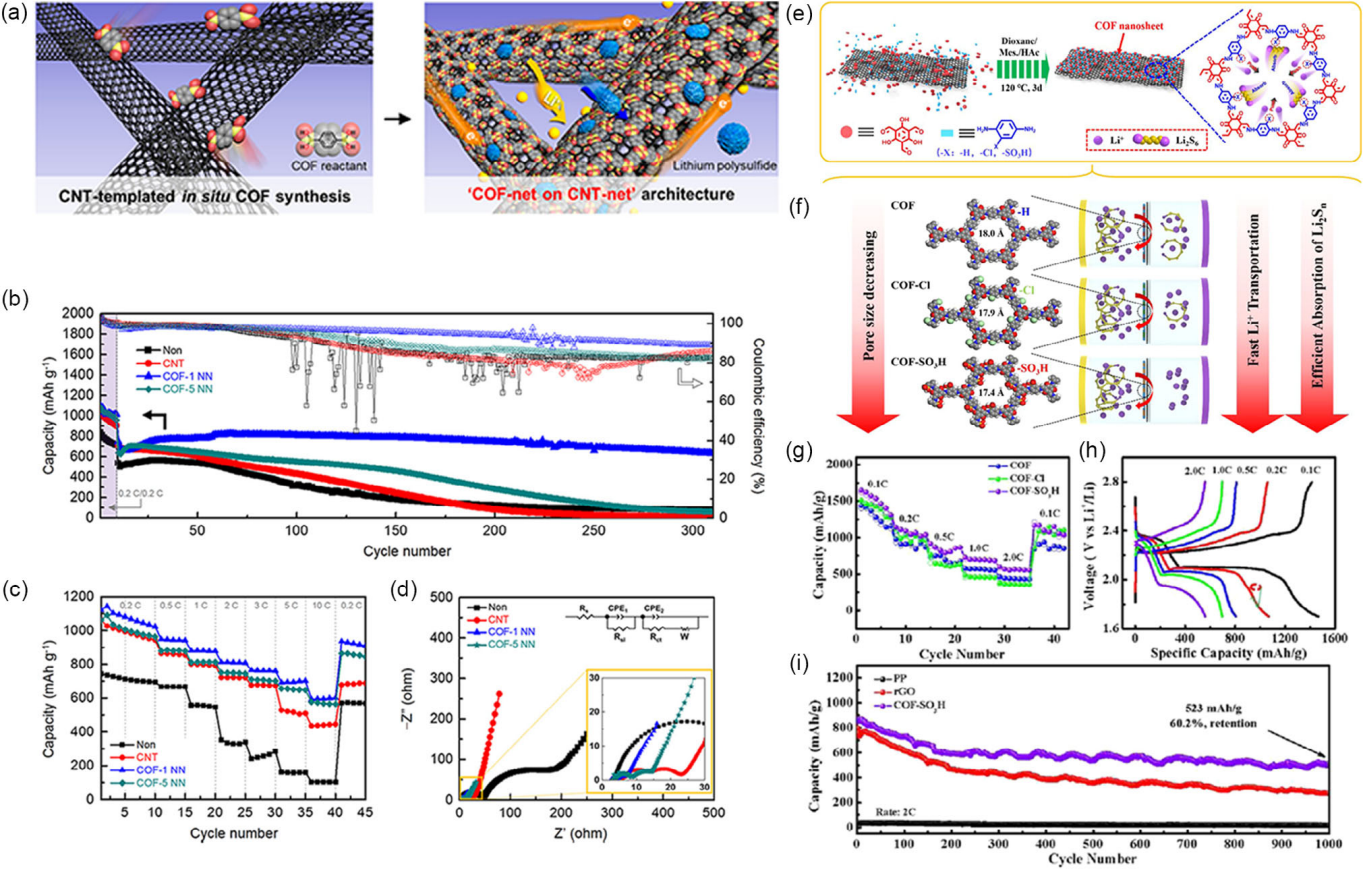
Li–S battery with COF‐1 NN and COF–TpPa–X@rGO (–X: –H, –Cl, –SO_3_H)‐modified separators and their performances. a) Schematic illustrations of the construction process and ion/electron‐transfer behavior of N–N interlayers. b) Cycling stability of batteries with carbon nanotube (CNT)‐, COF‐1 NN‐, and COF‐5 NN‐modified separators at 2C. c) Rate performance of Li–S batteries with CNT‐, COF‐1 NN‐, and COF‐5 NN‐modified separators from 0.2 to 10.0C. d) Electrochemical impedance spectra after 300 cycles. a–d) Reproduced with permission.^[^ ^57^ ^]^ Copyright 2016, American Chemical Society. e) Schematic diagram of the fabrication of COF–TpPa–X@rGO (–X: –H, –Cl, –SO_3_H). f) The effect of side chain X (–X: –H, –Cl, and –SO_3_H) in pores of the three kinds COFs for Li^+^ transport and PSs adsorption. g) Rate capability of battery using different separators. h) Discharge/charge curves for COF–SO_3_H@rGO‐separator‐based battery. i) Cyclic stability of Li–S battery with rGO/COF–SO_3_H, rGO, and polypropylene (PP) separators at 2C. e–i) Reproduced with permission.^[^ ^60^ ^]^ Copyright 2022, American Chemical Society.

According to the timeline as shown in **Figure** **2**, remarkable progress in the modification of S‐based battery separator by COFs has been achieved in recent years. In 2016, Lee and colleagues have synthesized a hybrid structure of microporous COF network on mesoporous carbon nanotube (CNT) network, which can serve as a kind of porous chemical trap for PSs (**Figure** **3**a).^[^ ^57^ ^]^ A self‐supporting COF–net on CNT–net interlayer (i.e., NN interlayer) is prepared by in situ synthesis of COF on CNT template. As model systems, two types of COFs (COF‐1 and COF‐5) are chosen to investigate their battery performances. Among them, the COF‐1 NN interlayer can efficiently facilitate the selective deposition or dissolution of electrically inert Li_2_S due to its recherche microporous structure. Thus, the COF–NN interlayer gives a high cycling performance (capacity maintained at 84% after 300 cycles at 2C current) (Figure 3b) and rate performance (Figure 3c). By testing the electrochemical impedance after cycling, the superior cyclability of battery utilizing the COF‐1 NN interlayer is demonstrated (Figure 3d). Subsequently, studies have been conducted on the introduction of sites with high electron affinity such as N and O into COF structures. Li et al. demonstrated an innovative strategy with a COF including the pyridine groups as the separator modification layer,^[^ ^58^ ^]^ in which the COFs perform multifarious roles: the pyridine nitrogen could form a Li bond through the dipole–dipole interactions with Li^+^; the keto groups of TP would improve the ability to adsorb PSs, and the pore channels of COFs could facilitate Li^+^ transfer. Additionally, the pyridine units could act as the catalysis centers for the conversion of PSs, which can serve as the redox‐active group. The battery with the COFs‐modified separators displays outperformance in cycling stability (after 250 cycles at 1C, 826 mAh g^−1^).

Subsequently, Wang and co‐workers studied the performance of sulfonate‐rich, monosulfonate, and non‐sulfonate COFs for Li–S battery separator modification.^[^ ^59^ ^]^ The dual‐sulfonated COF (SCOF‐2) has the benefits of concentrated negative charge, improved interlayer spacing, and narrow bandgap, which concurrently promotes Li^+^ transfer and mitigates the occurrence of dendrites. In addition, the SCOF‐2 can inhibit PSs shuttling by chemical trapping and electrostatic repulsion. Hence, the battery with SCOF‐2‐based separator displays a low deterioration rate after 800 cycles of 0.047% per cycle. Then, in 2022, Lu et al. revealed the impact of various functional groups on Li–S battery performance and its principles in their manuscript (Figure 3e). They prepared COF–TpPa–X@rGO (rGO: reduced graphene oxide; –X:–H,–Cl,–SO_3_H) nanosheet hybrids with different functions by changing the monomers.^[^ ^60^ ^]^ According to Figure 3f, the presence of functional groups may control the adsorption of PSs and Li^+^ transmission. In addition, the structure of the electron cloud in COFs is altered by side chain groups, which would impact the properties such as electrical conductivity. Specifically, the properties of COF–SO_3_H@rGO separator outperform the pure rGO, COF@rGO, and COF‐Cl@rGO separators both in terms of cyclic capacity and rate performance (Figure 3g,i). Even at 2 C, the typical two‐platform voltage curves show low polarization and high capacity (Figure 3h). The results show that the COF–SO_3_H@rGO separator presents high cycling performance (after 1000 cycles at 2C, 60.2% retention, Figure 3i) and specific capacity (1163.4 mA h g^−1^ at 0.2C). In addition, researchers have also explored other functional groups and representative COFs are listed in other sections and described in conjunction with their performances.

### 2.2 Preparation Method of COFs‐Modified Separators

**Figure 4:**
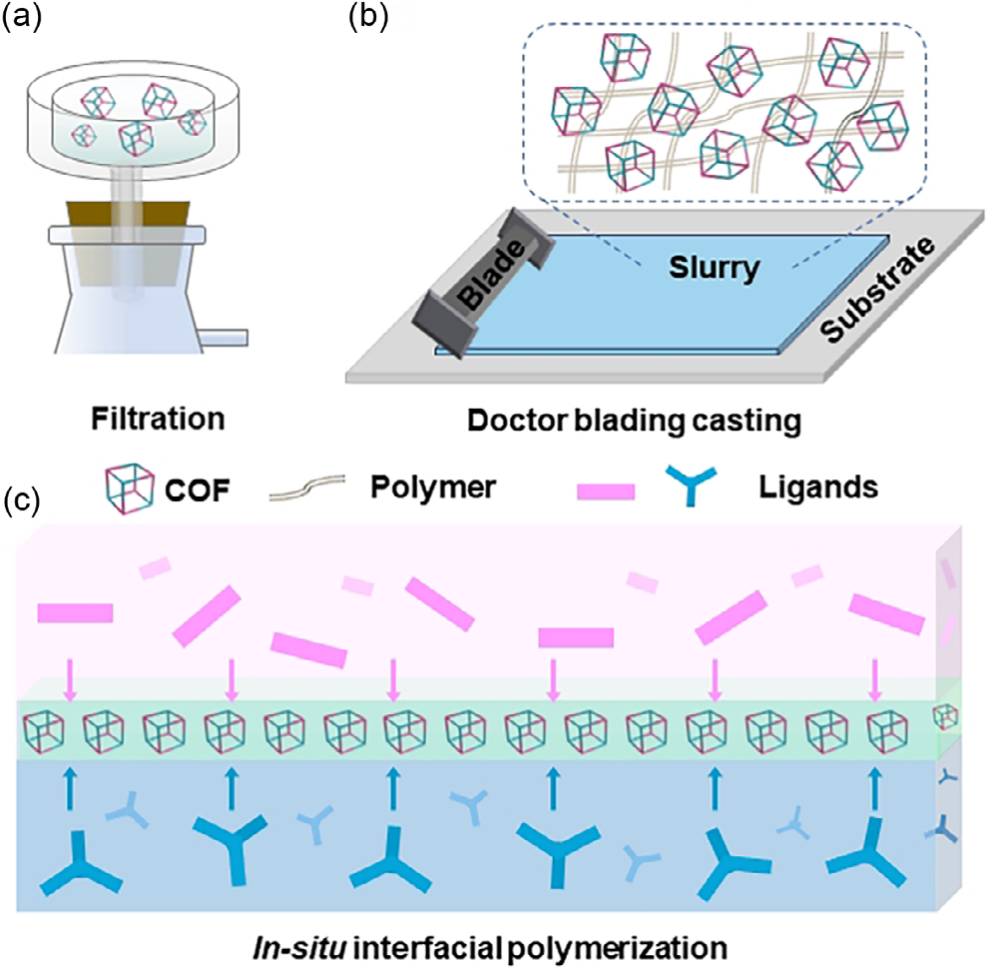
Different preparation methods of COFs‐modified separator. a) Filtration. b) Doctor blading methods. c) In situ interfacial polymerization.

Up to date, several approaches have been reported for the construction of COFs‐modified separators, containing vacuum filtration, doctor blade casting, and interfacial in situ polymerization, etc. (**Figure** **4**).^[^ ^18^, ^23^, ^61^ ^]^ Among them, vacuum filtration is a simple and common strategy for preparing membranes at room temperature, which can control the loading of COFs and the thickness of modification layer (Figure 4a). For example, Negishi et al. optimized the loading thickness of the modified layer by using different masses (5–10 mg) of COF@graphene (COF@G) composite dispersed in a specific volume of 1‐methyl‐2‐pyrrolidone and also explored the effect of diverse ratios on the electrochemical properties when the materials are compounded.^[^ ^62^ ^]^ After optimizing the conditions, the COF@graphene‐modified separator using 10 mg and ratio of COF:graphene (1:1) displays an initial capacity of 1489.8 mA h g^−1^ at 0.2 A g^−1^ and stable cycling performance together with excellent rate performance. However, in this method, the COFs dispersion, morphology tuning, or processing techniques affect the homogeneity of the modified layer, which is closely related to the battery performance. Additionally, the vacuum filtration strategy is subjected to rigor through the experimental apparatus and is therefore difficult in scale‐up production.

In addition to the vacuum filtration method, the direct casting method through the doctor blade is another method for the preparation of separators, which is also a commonly applied technique in industry (Figure 4b). For instance, an anisotropically hybridized separator (CPM: COF–IL/PP/MOF) using ionic liquid (IL)‐modified porphyrin‐based COF and catalytically conductive MOF was constructed and applied in Li–S batteries by Lan's group.^[^ ^63^ ^]^ Using a doctor blade, the COF–IL suspension with PVDF–HFP was cast onto commercial PP. The fabrication conditions (i.e., loading of COF–IL and layer thickness) in CPM can be easily altered based on the doctor blading procedure, allowing for the preparation of a variety of CPM separators to study their battery properties. At a current density of 0.2C, the cycling capabilities of battery applying CPM separators with various casting thicknesses (50–750 μm) and COF–IL loadings (10–30 wt%) have been assessed. Thus, the optimal conditions are selected (250 μm and 20 wt% loading).

However, the methods mentioned earlier still have some problems, such as interface impedance and weak interaction between the interface and modified layers, that might cause the modified layer to be detached and peeled off easily. Hence, it is essential to explore other methods to conquer these obstacles and some pioneering techniques like in situ interface polymerization have been proposed. For instance, an in situ interfacial polymerization strategy has been proposed by Qu et al. to synthesize a kind of TpPa–SO_3_H@PP separator (Figure 4c).^[^ ^61^ ^]^ The different ligands are first dissolved in the organic and aqueous phases, and then a PP separator, acting as a semipermeable membrane, is immobilized at the solution interface. PP separators with deposited COFs membranes are obtained by dropping the aqueous phase solution onto the separator layer. This novel synthesis method allows in situ growth of commercial PP separators by growing them internally and externally without obvious increase in volume and mass. In this regard, the battery with TpPa–SO_3_H@PP separator demonstrates excellent cycling performance of 863.97 mAh g^−1^ at 1C initially and maintains at 645.62 mAh g^−1^ after 500 cycles (0.05% fading per cycle). Therefore, in situ interfacial polymerization could lower the interfacial resistance and lead to the uniform performance of the modified separator with industrial production potential, but it is still limited in the types of COFs that can be synthesized.

### 2.3 Application of COFs in Li–S Battery Separators

Among the metal–sulfur battery systems, the Li–S battery is intensively researched since 1970s, and there are thousands of publications on Li–S batteries every year.^[^ ^17^, ^18^, ^19^, ^20^ ^]^ Specifically, COFs as a kind of promising functional materials have also been explored for the utilization of Li–S battery separators over the past few years.^[^ ^64^ ^]^ In this section, we will summarize the applications of different COFs in the modification of Li–S battery separators including their related battery performances in **Table** **2**. We have made a brief classification about the COFs‐modified separator and will discuss detailedly in the following sections.

**Table 2:** A summary of COFs‐modified separator performance for Li–S batteries

	Substrate separator	Modified material	Method	S content [mg cm^−2^]	Cycling performance [mA h g^−1^]	Reference
1	PP separator	COF–TpPa–SO_3_H@rGO	Casting	0.9	523 (1000 cycles, 2 C)	[60]
2	Celgard 2400	TAPP–ETTB@GO	Vacuum filtration	1.5–2.0	920 (400 cycles, 0.2 A g^−1^)	[62]
3	Celgard 2400	TpPa–SO_3_H@PP	In situ interfacial polymerization	1.0	645.62 (500 cycles, 1C)	[61]
4	Ceramic	DMTA‐COF	Casting	1.5	1000 (100 cycles, 0.5C)	[65]
5	Celgard	CTFs	Casting	2.0	700 (800 cycles, 1C)	[68]
6	Celgard 2500	CTF	Vacuum filtration	/	684 (400 cycles, 1C)	[69]
7	Celgard 2400	PyBBT‐COF	Vacuum filtration	1.0	905 (100 cycles, 0.2C)	[70]
8	Celgard	TP–BPY‐COF	Casting	1–1.5	826 (250 cycles, 1C)	[58]
9	Celgard 2325	SCOF‐2	Casting	1.2–2.0	497 (800 cycles, 1C)	[59]
10	Celgard PP/PE/PP	TpPa–SO_3_Li/CNT	Vacuum filtration	1.5	482 (400 cycles, 4C)	[71]
11	Celgard 2325	SCOF	Casting	1	750 (120 cycles, 0.5C)	[72]
12	PP separator	4F‐COF	Casting	0.8–1	873.1 (100 cycles, 0.2C)	[73]
13	Celgard 2400	CB‐COF	Casting	2.0	569 (1000 cycles, 1C)	[74]
14	polyethylene	COF‐1 on CNT	Vacuum filtration	1.1	84% (300 cycles, 2.0C)	[57]
15	Ceramic	CNT@DMTA‐COF	Casting	2.0	621 (500 cycles, 1 A g^−1^)	[75]
16	Celgard 2500	Py–DPP‐COF@CNT	Casting	1.2–2.0	584 (1000 cycles, 1C)	[32]
17	Celgard 2400	C@COF	Vacuum filtration	1.2–1.5	655.7 (500 cycles, 1C)	[76]
18	Celgard	HUT9@CNT	Casting	1.1	750 (500 cycles, 1C)	[77]
19	Celgard	COF‐rGO	Vacuum filtration	1.0–1.5	1169.4 (50 cycles, 0.1C)	[78]
20	Celgard	Li‐CON	Vacuum filtration	1.0–2.0	645 (600 cycles, 1C)	[79]
21	Celgard 2400	Ti_3_C_2_@iCON	Vacuum filtration	1.2	706 (2000 cycles, 2C)	[80]
22	Celgard 2500	TpPa–SO_3_H	Vacuum filtration	1.2	494 (500 cycles, 1C)	[81]

In fact, the demands for the separators are diversified owing to the different conditions on both sides of the electrode. For the separators orienting S cathode, the PSs adsorption/catalysis should be considered first. At the same time, an anodic interlayer should be electron insulated while preserving Li^+^ transmission and suppressing dendrite growth. Specifically, the porous and regular structures of COFs are beneficial for the ion/electrolyte transport and can be applied to effectively induce the uniform metal‐ion deposition and reduce dendrite generation. Meanwhile, COFs containing PSs‐adsorption/catalytic conversion sites can be applied to efficiently prevent the shuttling effect and enhance the battery performance on the S‐based cathode side. On this basis, we will classify the COFs‐modified separators into two categories (modified separator facing S‐based cathode or facing Li anode), and introduce related works in the following two sections.

#### 2.3.1 COFs‐Modified Separator Facing S Cathode Side

The main reason affecting the performance of Li–S cells is the shuttling effect of PSs, and thus adsorbing PSs or catalyzing long‐chain PSs into short‐chain PSs are the strategies to effectively suppress the shuttling effect.^[^ ^13^ ^]^ COFs can not only block PSs shuttling by a dense network, but also expand the density of adsorb/catalyze PSs sites by predesigned geometry and uniformly dispersed polar bonds, thus effectively inhibiting the shuttling effect.

**Figure 5:**
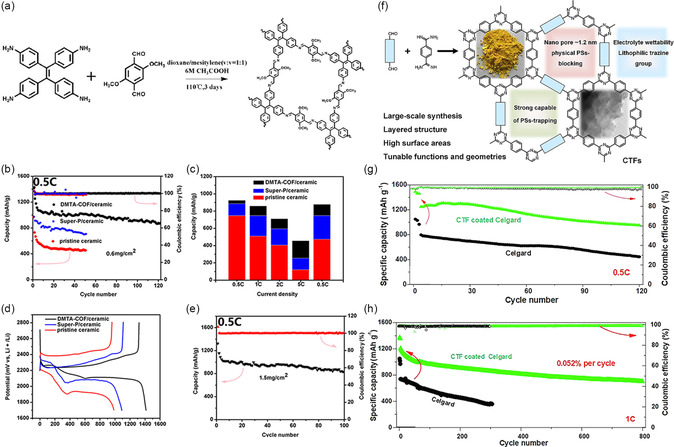
DMTA‐COF‐ and CTF‐modified separators and their battery performances. a) Schematic synthesis and structure of DMTA‐COF. b) Comparison of the cycling stability of the battery with pristine ceramic, super‐P/ceramic and DMTA‐COF/ceramic separator at 0.5C. c) Column diagram of pristine ceramic, super‐P/ceramic and DMTA‐COF/ceramic separator at different rates. d) Galvanostatic charge–discharge profiles of pristine ceramic, super‐P/ceramic and DMTA‐COF/ceramic separator at 0.5C. e) Electrochemical performance of battery using DMTA‐COF/ceramic separator with 1.5 mg cm^−2^ S loading at 0.5C. a–e) Reproduced with permission.^[^ ^65^ ^]^ Copyright 2018, American Chemical Society. f) Schematic diagram of the CTF‐coated interlayer and its advantages in Li–S battery. g) Electrochemical performance of the batteries with Celgard and CTF–Celgard at 0.5C and 30 °C. h) Long life cycle test of batteries with Celgard and CTF–Celgard at 1C and 30 °C. f–h) Reproduced with permission.^[^ ^68^ ^]^ Copyright 2019, Elsevier.

To prevent the shuttling effect, the design of pore channels for COFs is very important. For instance, an AB‐stacking COF (DMTA‐COF: 2,5‐dimethoxy‐1,4‐dicarboxaldehyde (DMTA)‐4,4′,4″,4‴‐(ethene‐1,1,2,2‐tetrayl)tetraaniline‐COF) with 0.56 nm pore size that could effectively mitigate the shuttling of PSs (0.51–0.68 nm) has been prepared by Cai and colleagues (**Figure** **5**a).^[^ ^65^ ^]^ Using this porous COF as a coating for ceramic separators, the corresponding cells initially have a capacity of 1415 mA h g^−1^ at 0.5C and preserve 1000 mA h g^−1^ after 100 cycles (Figure 5b). As the nanopores in the DMTA‐COF can prevent the PSs on the separator, the shuttling effect and the decrease of S are reduced. Compared to the original ceramic and the Super‐P‐modified, the performance of the DMTA‐COF‐based separator is significantly improved (Figure 5c–e). To demonstrate the nanopore advantage, a TAPB–PDA‐COF‐based separator (synthesized from 1,3,5‐tris(4‐aminophenyl)benzene (TAPB) and terephthalaldehyde (PDA)) (pore size: 2.52 nm) is also prepared. In contrast, battery with TAPB–PDA‐COF‐based separator initially provides a capacity of 1065 mA h g^−1^ at 0.5C, and decreases to only 525 mAh g^−1^ after 100 cycles. This is a consequence of the pores in the TAPB–PDA‐COF might be unsuitable to efficiently accommodate the PSs.

Except for the design of pore channels, researchers prefer to introduce functional groups in the structures to effectively inhibit shuttling effect or promote ion conduction. The common functional groups that can possess effect on the interaction with PSs in COFs include electron‐rich atoms (e.g., N and O, etc.), redox active sites (e.g., –C = O, –C = N–, C = C, etc.), anionic groups (–SO_3_H), and multi‐electron macrocycles groups (e.g., porphyrins) would contribute to the adsorption/catalysis of PSs.^[^ ^59^, ^66^, ^67^ ^]^

For example, Ye et al. demonstrated the 2D covalent triazine framework (CTF) including benzene derivatives and triazine rings with the functions of capturing PSs chemically, and lithiophilic interaction of the heteroatoms could result in reduced PSs shutting, high utilization of S, the protection of Li anode, and low self‐discharge (Figure 5f).^[^ ^68^ ^]^ The multifunctional CTFs‐based separators have the capacity to physically interact with PSs due to the adsorption of pyridinic/pyrrolic nitrogen with PSs. Moreover, CTFs have high porosity and excellent wettability of electrolyte, and possess abundant lithiophilic triazine groups that aid in Li^+^ transfer inside their host framework. It is proved that the battery with CTFs‐coated separators (≈0.14 mg cm^−2^) can exhibit a high initial discharge capacity (1249 mAh g^−1^ at 0.5C) (Figure 5g), while showing a rate performance (802 mAh g^−1^ at 2C), good capacity retention (decay rate of 0.052% per cycle after 800 cycles at 1C) (Figure 5h), remarkable anti‐self‐discharge ability and excellent Li‐anode‐protection capacity.

Subsequently, Zhang et al. modified the general Li–S battery separator using a CTF‐type COF.^[^ ^69^ ^]^ The CTF/CNT composite could offer a regular channel for quick Li^+^/electronic transport and exhibits potent PSs‐adsorption properties. Using the CTF/CNT/Celgard separator, the rate capability and cycle properties of the battery are obviously promoted. At 1C, the battery exhibits a high capacity of 684 mAh g^−1^ after 400 cycles. As comparison to Celgard separator, the battery utilizing the CTF/CNT/Celgard separator shows better performance even at a 2 mg cm^−2^ loading of S. Benzobisthiadiazole (BBT) with electron‐rich N/S possesses a strong electron‐withdrawing property. The unique electron affinity allows the BBT‐derived PyBBT‐COF to capture PSs for redox conversion at the separator of a Li–S battery. An imine‐linked tetragonal 2D COF was created by Guo et al. by combining a BBT‐derived building block with a pyrene‐based unit.^[^ ^70^ ^]^ Due to the combination of highly available pore channels and high electron affinity, the Li–S cell separator modified with COFs could conquer the shuttling effect. In the aspect of cycling performance at 0.2C, the PyBBT‐COF‐based Li–S battery provides a reversible capacity of 1249 mA hg^−1^ and maintains at 905 mA hg^−1^ after 100 cycles. At 1C, the PyBBT‐COF–based battery also achieves a reversible capacity of 948 mAh g^−1^ and outstanding cycling performance (0.083% fading per cycle after 500 cycles).

**Figure 6:**
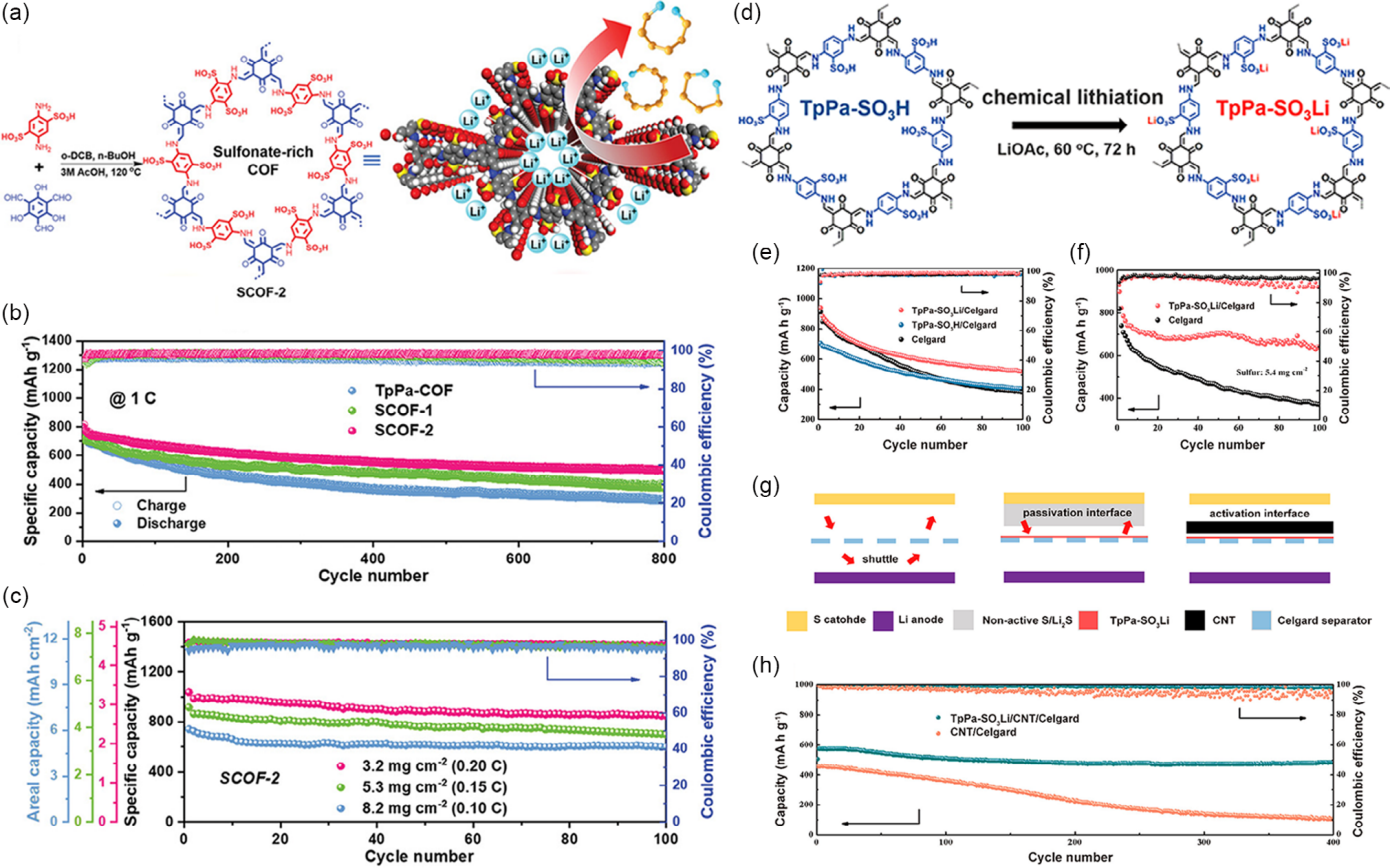
Li–S battery with dual‐sulfonated COF (SCOF‐2) and TpPa–SO_3_Li‐modified separators and their performances. a) Schematic synthesis of the sulfonated COFs (SCOF) and its application in Li–S battery. b) Long life cycling test of batteries with different separator over 800 cycles at 1C. c) Cycling test of batteries with SCOF‐2‐based separator under various S loading of 3.2, 5.3, and 8.2 mg cm^−2^, respectively. a–c) Reproduced with permission.^[^ ^59^ ^]^ Copyright 2021, Wiley‐VCH. d) Schematic of the Li–S cell with the TpPa–SO_3_Li/Celgard separator. e) Cycling test of batteries applying TpPa–SO_3_Li/Celgard, TpPa–SO_3_H/Celgard, and pristine Celgard at 0.2C. f) Cycling test of the batteries applying TpPa–SO_3_Li/Celgard and pristine Celgard under 5.4 mg cm^−2^ S loading at 0.2C. g) Schematic illustration of the batteries with TpPa–SO_3_Li/CNT/Celgard, TpPa–SO_3_Li/Celgard, and pristine Celgard. h) Cycling test of the batteries with TpPa–SO_3_Li/CNT/Celgard and CNT/Celgard at 4C. d–h) Reproduced with permission.^[^ ^71^ ^]^ Copyright 2021, American Chemical Society.

Furthermore, the introduction of some anionic groups (e.g., sulfonate (SO_3_^−^), into the COFs backbone, can efficiently adjust the Li flux and achieve stable long‐term Li plating/exfoliation at high current densities. Simultaneously, it can also inhibit PSs shuttling due to its electrostatic repulsive effect on PSs anions. For instance, Wang and his co‐workers have constructed a sulfonate‐rich SCOF‐2 for separator modification (**Figure** **6**a).^[^ ^59^ ^]^ The results show that SCOF‐2 is more electronegative and has wider interlayer spacing in comparison to sulfonate‐free/monosulfonate COFs, which could promote Li^+^ transport and mitigate the growth of dendrites. The self‐discharge behavior of SCOF‐2 is suppressed by its small bandgap and strong interactions with PSs, which is revealed by density‐functional theory (DFT) calculation and other experiments. The improved battery provides 0.047% within 800 cycles and offers excellent self‐discharge resistance through a low‐capacity decline of 6.0% per cycle (Figure 6b). Furthermore, the cell shows ≈80% capacity retention after 100 cycles even under 3.2–8.2 mg cm^−2^ S loading and a lean electrolyte (5 μL mg^−1^), demonstrating possibility for practical applications (Figure 6c). By substituting Li for H in the intrinsically ordered pores of COFs, aligned nanochannels for Li conduction are created, which increases the transfer rate of Li. In addition, Sun and colleagues have synthesized TpPa–SO_3_Li layer onto the Celgard separator (Figure 6d).^[^ ^71^ ^]^ At 0.2C, the battery can accomplish a high capacity (822.9 mA h g^−1^) and a high retention rate (78% over 100 cycles) with a total mass S loading of 5.4 mg cm^−2^ using the TpPa–SO_3_Li layer (Figure 6e,f). The advantage that TpPa–SO_3_Li layer can successfully boost the Li^+^ transfer while limiting PSs diffusion through the electrostatic contact due to the existence of ordered channels and consecutive sites of negative charge (Figure 6g,h). Moreover, Zhang et al. reported a sulfonated COF (SCOF) as a modified layer for the separator.^[^ ^72^ ^]^ The lithiated sulfonate groups may facilitate Li^+^ transportation and decrease the coating‐induced energy barrier of ion transportation. After 120 cycles at 0.5C, the battery with the SCOF‐modified separator displays a capacity retention of 81.1%. It also shows a modest capacity fading over 600 cycles at 1C of 0.067% per cycle. In addition, even at 2C, the battery exhibits a superior reversible capacity of 576 mAh g^−1^.

**Figure 7:**
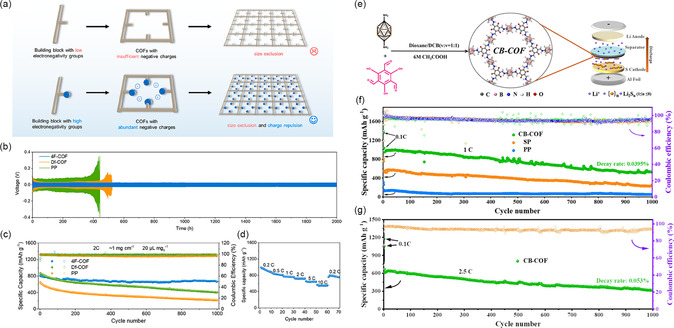
Li–S battery with fluorinated COF (4F‐COF)‐ and carbon‐alkyl COF (CB‐COF)‐modified separators and their performance. a) Construction of COFs (4F‐COF) with electron‐negative building components for size‐exclusion and charge‐repulsion membranes. b) Cycling performance of Li/Li symmetric cells with 4F‐COF, Df‐COF (Df‐COF was synthesized with low‐electronegativity 1,4‐diformylbenzene as the aldehyde linker for comparison), and PP separators at 1 mA cm^−2^ and 1 mA h cm^−2^. c) Long‐term cycle test of Li–S battery with 4F‐COF, Df‐COF, and PP separators at 2C. d) Rate performance from 0.2 to 10C of Li–S battery with 4F‐COF/PP separator. a–d) Reproduced with permission.^[^ ^73^ ^]^ Copyright 2023, American Chemical Society. e) Schematic illustration of the synthesis of CB‐COF and composite separator. f) Cycling test at 1C of CB‐COF‐modified‐separator‐based Li–S battery. g) Cycling test at 2.5C of CB‐COF‐modified‐separator‐based Li–S battery for 1000 cycles. e–g) Reproduced with permission.^[^ ^74^ ^]^ Copyright 2021, American Chemical Society.

In addition to functional groups like electron‐rich atoms and SO_3_^−^, the introduction of F site in the COFs structure can also help to construct negative charge channels for regulating the ion migration behavior of Li–S battery. In 2023, Xie et al. constructed a nanofluidic membrane based on a fluorinated COF (4F‐COF) and employed in Li–S battery (**Figure** **7**a).^[^ ^73^ ^]^ Fluorine in the channels contributes to the construction of permselective nanofluidic channels. The nanofluidic separator based on 4F‐COF prevent PSs shutting while permitting Li^+^ transfer due to the elevated density of well‐organized and negatively charged ion channels. Specifically, the Li–S battery with 4F‐COF exhibits Li metal durability surpassing 2000 h at 1 mA cm^−2^ (Figure 7b), capacity decay of 0.018% per cycle after 1000 cycles at 2C (Figure 7c), and rate capability of 568 mA h g^−1^ at 10C (Figure 7d).

Furthermore, there are also some interesting works about introducing other active sites into COFs that can be functional units in Li–S battery separator. For example, Yu and co‐workers have synthesized an amphiphilic carbon‐alkyl COF (CB‐COF), and the CB building block acts as a 3D structure with various pro‐sulfur‐adsorption sites to capture PSs, which significantly increases the capture efficiency of PSs (Figure 7e).^[^ ^74^ ^]^ In addition to the developed channels for the transmission of Li^+^ and electrons, CB‐COF also contains a lot of active sites (C–O and B–H) to adsorb PSs. Both theoretical calculations and experimental results show that CB‐COF has an effective PSs‐adsorption capacity. This ability allows the separator with CB‐COF to effectively restrain the shuttling effect, leading to a long cycle life of 314 mA h g^−1^ with 1000 cycles at 2.5C (decay rate of 0.0395% at 1C over 1000 cycles) (Figure 7f,g). When the S loading is increased to 9 mg cm^−2^ at a low E/S ratio (6.37 μL mg^−1^) at 0.1C, it still provides an area capacity of 6.3 mAh cm^−2^ after 100 cycles.

**Figure 8:**
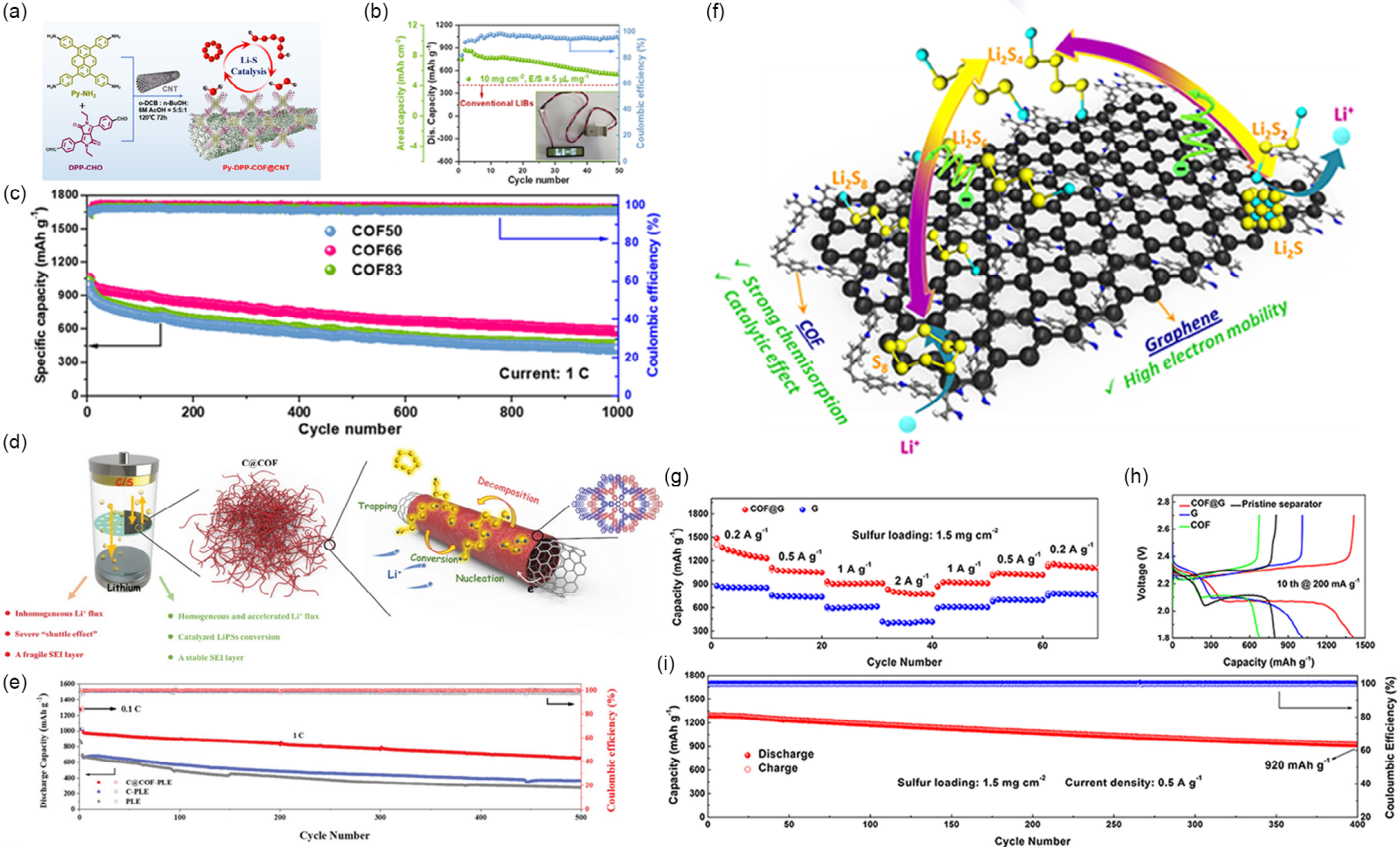
Li–S battery with COF@CNT and COF@graphene (COF@G)‐modified separators and their performance. a) Schematic diagram of the COF@CNT synthesis procedure and its catalytic application. b) Cycling test of battery with COF@CNT separator under 10 mg cm^−2^ S loading at 0.1C. c) Long‐term cycling test of battery with COF50‐, COF66‐, and COF83‐based separators, respectively. a–c) Reproduced with permission.^[^ ^32^ ^]^ Copyright 2021, American Chemical Society. d) Schematic images of working process with commercial separators and C@COF‐modified separators in Li–S battery. e) Cycling test of battery with C@COF‐based separators at 1C. d,e) Reproduced with permission.^[^ ^76^ ^]^ Copyright 2022, Wiley‐VCH. f) Schematic image of COF@G interlayer in Li–S battery. g) Rate capability of batteries with COF@G and G separators. h) Discharge/charge curves for the tenth cycles of batteries with COF@G, G, COF, and pristine separators at 0.2 A g^−1^. i) Cycling test of batteries with COF@G‐modified separators at 0.5 A g^−1^ over 400 cycles. f–i) Reproduced with permission.^[^ ^62^ ^]^ Copyright 2021, American Chemical Society.

For applications of COFs‐modified separators facing the cathode side, the conductivity of the material is also extremely important. Conductive structure smooths current distribution and makes sure that active components can be contacted. Meanwhile, conductive modification layer can also provide additional reaction sites for active S and reduce PSs release during the discharge process. Additionally, the electrons can effortlessly transform from electrode to separator, allowing for the realization of redox processes. Thus, the absorbed PSs could be used upon cycling, effectively improving the utilization of S. Traditionally, the conductivity can be enhanced by physical mixing (e.g., adding conductive carbon materials to the mixed slurry) during the battery processing. However, the mechanical mixing and ununiform dispersion caused by the particle aggregation of COFs might lead to partial aggregation of ions and electrons, thus leading to poor performance. Therefore, some researchers have discovered the hybridization of porous carbon materials (e.g., graphene and CNTs) with COFs through the in situ growth of COFs on the surface of conductive agents (**Figure** **8**).

CNTs are commonly used as conductive materials in Li–S battery. In 2020, Teng et al. have designed an innovative functionalized separator (COF–CNT–separator) to suppress the dissolved PSs by combining COFs with an interlude CNT network.^[^ ^75^ ^]^ Remarkably, it serves as both a house for PSs and an ionic sieve to effectively sieve PSs and contain them in the cathode area. After the first cycle, the battery has a 1068 mAh g^−1^ reversible capacity at 1 A g^−1^, and after 500 cycles, the capacity can be stabilized at 621 mAh g^−1^ (80% S content). Li–S batteries with a COF–CNT–separator can operate among the temperature range of 10–50 °C and exhibit high S utilization rates, outstanding rate, and cycle performance. As a result, the facile method of using a separator with a unique network to create a high‐performance Li–S battery is a strong contender.

Then, Chen and his co‐workers fabricated a kind of diketopyrrole (DPP)‐based COF separator (denoted as COF@CNT) through the in situ growth method (Figure 8a).^[^ ^32^ ^]^ By adding a suitable CNT concentration (66 wt%), the electrocatalytic activity can be maximized. DFT calculations and experiments demonstrate that the DPP unit can control PSs conversion by –C=O–/–C–O– bond. The Li–S battery achieves the target capacity of 8.7 mAh cm^−2^ with a S loading of 10 mg cm^−2^ and a lean electrolyte (E/S = 5) (Figure 8b), and displays a fading rate of 0.042% after 1000 cycles (Figure 8c).

Subsequently, Wu et al. have prepared a layered interlayer constructed from boroxylated COFs with high Li^+^ conductivity by similar COFs in situ growth strategy onto CNT (C@COF) (Figure 8d).^[^ ^76^ ^]^ Due to the rich heterogeneous interface between the interior conducting CNTs and the exterior COFs, the interlayer functions as both a physical barrier and a catalyst for PSs adsorption/catalysis, offering high Li^+^ conductivity (1.85 mS cm^−1^) and Li^+^‐transfer number (0.78). The battery with this catalytic interlayer exhibits a fading rate of 0.07% in 500 cycles at 1C and a lifetime more than 1000 cycles at 3C (Figure 8e). In 2022, Zhang et al. have synthesized a new disulfide‐bonded COF (HUT9) that can be generated on the surface of CNTs as a separator modification material in Li–S cell.^[^ ^77^ ^]^ DFT calculations and experiments show that the disulfide bond can efficiently trap soluble PSs while accelerating the conversion of PSs to Li_2_S/Li_2_S_2_ and improving S utilization. The polar groups of the COFs deliver chemisorption, while the interwoven network (physically bound) of HUT9@CNT synergistically inhibits the transport of PSs. After 500 cycles at 1C, the modified cell still retains 83% of its capacity (attenuation per cycle was 0.032%). To be more precise, the improved 2‐HUT9@CNT separator exhibits a 3.4 mAh cm^−2^ area capacity after 50 cycles at S loading of 4 mg cm^−2^ (S content: 80 wt%, E/S ratio: 10 mL g^−1^).

The combination of COFs with graphene has also received much attention due to conductive network of graphene can decrease the Li^+^‐transfer distance and promote the performance of COFs. For example, by depositing COFs onto graphene, Hu and colleagues have developed an innovative approach for easily anisotropic ordering of 2D COFs.^[^ ^78^ ^]^ With outstanding shuttling effect inhibition and adaptability to Li–S battery, the obtained membranes serve as ionic sieves leading to great cyclability. The battery with the COF–rGO membrane displays a capacity of 1386.9 mA h g^−1^ initially and maintains at 1169.4 mA h g^−1^ after 50 cycles. In addition, Sun and his group have constructed COF nanosheets assembled with graphene nanosheets (GNs) through the Schiff base reaction, providing a lithiated COF nanosheet (Li‐CON) containing rich functional units like triazole and phenolic, as well as ordered channels for the effective introduction of Li sites.^[^ ^79^ ^]^ COFs assembled with 2D GNs can form compact layers on commercial Celgard separators, in which GN can act as a conductive network to anchor PSs to decrease the interfacial resistance and lessen the Li^+^‐transfer distance. Li‐CON@GN cells have lower polarization (Δ*V*), smaller overcharge potential, and lower charge‐transfer resistance (*R*_ct_) compared to CON@GN cells and GN cells. After 200 cycles at 0.5C, the battery applying Li‐CON@GN still maintains at 750 mA h g^−1^, which is more superior to the other contrast samples. After 600 cycles, Li‐CON@GN cells exhibit a reversible capacity of 645 mA h g^−1^ with a fading rate of 0.057% per cycle. Furthermore, COF@G composites with PSs blockers and conversion catalysts have also been prepared by Negishi et al. (Figure 8f).^[^ ^62^ ^]^ The crystalline TAPP–ETTB COF (synthesized from 5,10,15,20‐tetrakis(4‐aminophenyl)porphyrin (TAPP) and from 4,4′,4″,4‴‐(ethene‐1,1,2,2‐tetrayl)tetrabenzaldehyde) (ETTB) is employed to have a regular pore geometry, which provides sufficient sulfophilic sites for strong chemisorption and catalysis of PSs. Meanwhile, graphene can achieve high electron transport, which enhances the redox kinetics of S. At 0.2 A g^−1^, the TAPP–ETTB@G separator‐based Li–S battery exhibits a high reversible capacity of 1489.8 mAh g^−1^ along with excellent cycling properties (920 mA g^−1^ after 400 cycles) (Figure 8i) and good rate capacity (827.7 mAh g^−1^ at a current of 2 A g^−1^) (Figure 8g,h).

**Figure 9:**
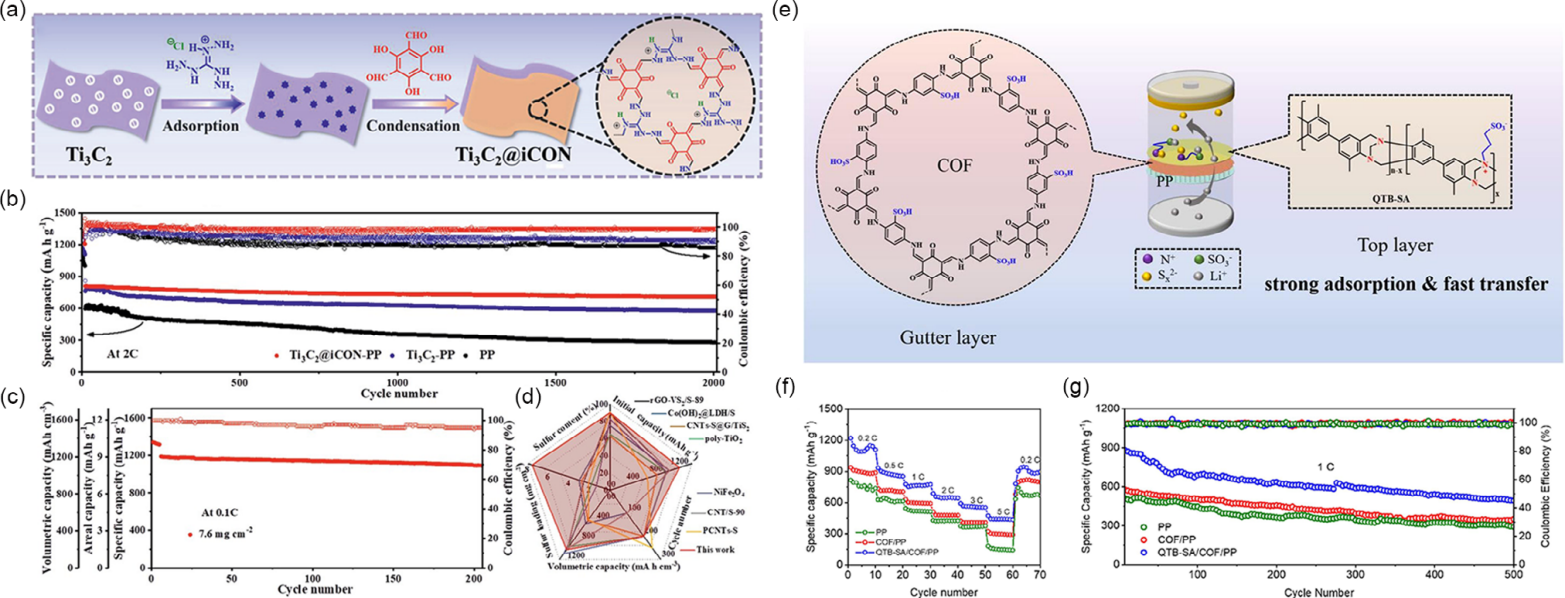
Li–S battery with COF/MXenes and COF/QTB‐SA composite–modified separators and their performance. a) Schematic diagrams for the preparation of Ti_3_C_2_@iCON. b) Long life cycle test of batteries with Ti_3_C_2_@iCON–PP, Ti_3_C_2_–PP, and PP at 2C. c) Cycling stability of Ti_3_C_2_@iCON–PP with 90% S content and 7.6 mg cm^−2^ S loading at 0.1C. d) Comparison of the electrochemical performance of Ti_3_C_2_@iCON–PP with reported materials. a–d) Reproduced with permission.^[^ ^80^ ^]^ Copyright 2021, Wiley‐VCH. e) Schematic diagrams for the QTB–SA/COF/PP composite separator. f) Rate‐capability performance of batteries with QTB–SA/COF/PP, COF/PP, and PP separator. g) Long life cycle test of batteries with QTBSA/COF/PP, COF/PP, and PP separator at 1C. e–g) Reproduced with permission.^[^ ^81^ ^]^ Copyright 2023, Elsevier.

MXene, a kind of promising and functional materials with excellent conductivity, amphiphilic interactions with PSs, and catalytic effects, present much potential in Li–S battery. In 2021, Li *et al.* have prepared the modification layer of PP separator involving Ti_3_C_2_ nanosheets with guanidinium‐based iCON uniformly loading (Ti_3_C_2_@iCON) (**Figure** **9**a).^[^ ^80^ ^]^ Innovating platforms are provided by the synergetic effects of Ti_3_C_2_ and iCON, which can speed up the redox dynamics of PSs and enable efficient conversion of PSs. The Li–S battery applying Ti_3_C_2_@iCON coating layer displays a low capacity deterioration (0.006% per cycle in 2000 cycles at 2C) (Figure 9b). The separator is still effective at 7.6 mg cm^−2^ S loading and 90 wt% S content, with areal, reversible, and volumetric capacities of 9.01 mAh cm^−2^, 1186 mAh g^−1^, and 1201 mAh cm^−3^ at 0.1C, respectively (Figure 9c,d).

Except for the carbon materials, polymer materials are also one of the effective materials for combining with COFs to enhance performance. Li et al. found that a novel zwitterionic microporous polymer (QTB–SA) was an intriguing option for advanced Li–S battery because it could accomplish decent Li^+^ transfer and outstanding ion‐sieving effect (Figure 9e).^[^ ^81^ ^]^ By spin‐coating, they constructed a QTB–SA/COF/PP separator, which demonstrated quick Li^+^ conduction as well as efficient immobilization and catalytic conversion of PSs by integrating the microporosity of polymer and COFs and the zwitterionic properties of polymer. The experimental results present that the high prolonged cycling behavior of battery applying QTB–SA/COF/PP separator (0.087% capacity degradation per cycle at 1C after 500 cycles) and excellent rate performance (Figure 9f,g). Additionally, the Li–S battery can reach an areal capacity of 3.4 mAh cm^−2^ after 100 cycles at 4.5 mg cm^−2^ S loading.

#### 2.3.2 COFs‐Modified Separator Facing Li Anode Side

An essential prerequisite for ensuring the safety of the battery is the protection of the metal anode.^[^ ^13^, ^14^ ^]^ Due to the theoretical capacities and low potentials, Li is viewed as a potential material of anode for the next‐generation batteries. Nevertheless, inhomogeneous Li deposition and the appearance of unregulated Li dendrites lead to rapid capacity fading, low Li utilization, and poor cycling performance.^[^ ^16^ ^]^ The introduction of COFs may suppress these problems based on the following considerations: 1) the COFs with uniformly arranged channels may accelerate the deposition kinetics and produce uniform electric‐field distribution and 2) COFs with designable structure and imparted functional groups can provide Li‐adsorption sites and possess strong interfacial interaction with Li anode surface.

Cai et al. demonstrated that aromatic functional groups in COFs can guarantee the uniformly transfer of Li^+^.^[^ ^82^ ^]^ To protect the anode, the introduction of lithiophilic aromatic groups in PA‐COF coating layer could interact with Li^+^, decrease Li^+^ solvation, and promote uniform and quick Li^+^ conduction. Even at 20 mA cm^−2^, the Li^+^ can still be consistently deposited and stripped with the PA‐COF‐based separator in Li–Li symmetrical cell. In addition, Feng and his colleagues have designed the lipophilic COFs with triazine rings and carbonyl groups for Li metal battery interlayer.^[^ ^83^ ^]^ The periodically arranged subunits with numerous functional groups in lipophilic COFs are beneficial to ensure smooth Li deposition, consistent Li^+^ efflux distribution, and less Li dendrite growth. With extremely low polarization voltages of 0.5 mA cm^−1^ (12 mV) and 1.0 mA cm^−1^ (14 mV), respectively, the symmetric battery with COF interlayers exhibits exceptional cycling stability for over 2450 and 1000 h. The full cells coupled with S cathodes exhibit outstanding energy density and rate performance.

Specifically, separators that can possess synergistic effect to simultaneously conquer the bottlenecks of the both electrode sides would be much desirable for the progress of powerful separator to satisfy the high requirement of S‐based battery separators. Lan et al. reported an anisotropically hybridized separator (CPM) based on catalytically conductive Ni_3_(HITP)_2_ and porphyrin‐based COF modified with IL (COF‐366–OH–IL) for Li–S battery.^[^ ^63^ ^]^ DFT calculations and adequate characterizations demonstrate that CPM has anisotropic effects on both the cathodic (e.g., PSs adsorption/catalysis) and anodic sides (e.g., Li^+^ transfer, anode protection, and PSs adsorption) of the cell. Notably, the battery based on obtained separator has an ultralow polarization voltage (30 mV), high Li^+^‐transference number (t_Li+_ = 0.8), high initial specific capacity (921.38 mAh g^−1^ at 1C), and excellent cycling performance, which is superior to PP‐ and monolayer‐modified separators.

### 2.4 COFs‐Based Separator for Other S‐Based Batteries

To date, there have been numerous breakthrough studies concentrating on the advancement of Li–S batteries.^[^ ^14^, ^16^ ^]^ Nevertheless, owing to the restricted supply of Li sources and the expected price increase, using Li‐metal anodes may become problematic in the near future. Researchers have explored a variety of alternative battery systems connecting the high‐capacity S cathode with various metallic anodes (e.g., Na, K, Mg, and Ca, etc.) on account of the progress and advancement made in Li–S batteries.^[^ ^4^, ^10^ ^]^ Nevertheless, there are few works about COFs‐based separators in these systems at current stage. Based on our research of the published works, we find that the research interest of scientists mainly focuses on S‐based battery systems like Li–SeS_2_ and Na–S, and there remains much work to be carried out. Therefore, this part will briefly introduce the recent work on Li–SeS_2_ and Na–S battery, and we hope it will give new insight for the development of COFs in these alternative S‐based battery systems.

In contrast to Li, Na is gaining more and more attention in the battery field due to its more abundant reserves. Na–S battery is remarkable for the superhigh energy density (1230 Wh kg^−1^), and low cost of Na when compared with Li–S battery.^[^ ^84^ ^]^ For stationary energy‐storage applications, high‐temperature Na–S battery (300–350 °C) has been operated as commercial battery system. Nevertheless, the high operating temperature would limit its widespread use, thus room‐temperature Na–S batteries are gradually investigated by the research community.^[^ ^85^ ^]^ At room temperature, the drawbacks of the Na–S battery are more serious than that of the Li–S battery due to the difference between Na and Li: 1) Na is substantially more active than Li, thus the dendritic formation of Na anode is much more severe than Li anode^[^ ^15^ ^]^; 2) Na polysulfides have a worse shuttling impact than Li polysulfides caused by its higher solubility in liquid electrolytes^[^ ^86^ ^]^; 3) compared to a Li–S battery (80% volume expansion), the volume expansion of cathode in Na–S battery can reach up to 170%^[^ ^87^ ^]^; and 4) Na^+^ exhibits slower charge transmission and electrochemical reaction kinetics as well as increased cell polarization due to their larger radius and lesser mobility when compared to Li^+^. Considering the modifiable and well‐defined structures with high porosity of COFs, precisely designed COFs might serve as functional separators to promote the Na–S battery performance and related works will be listed in the following part.

**Figure 10:**
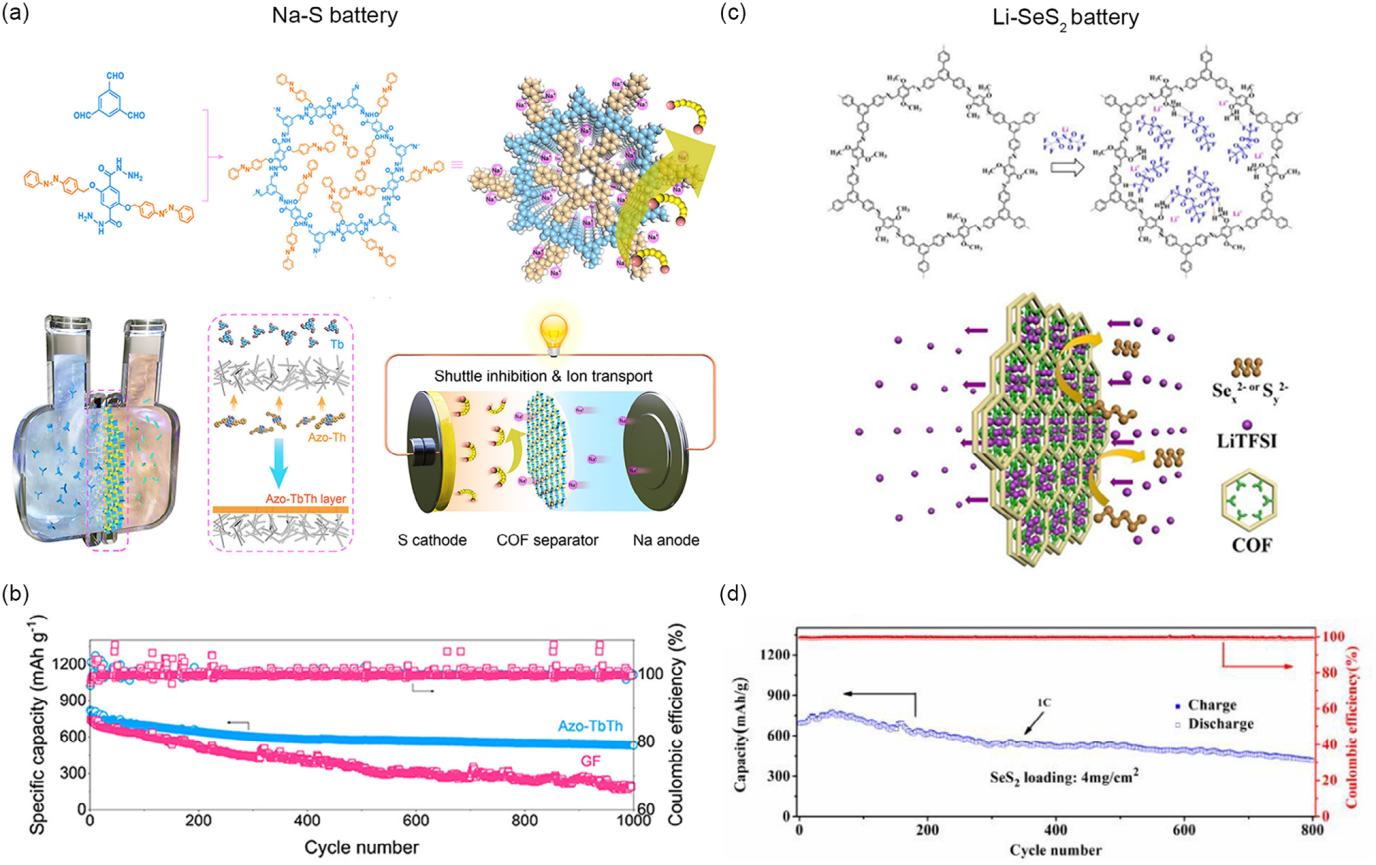
Schematic illustration and performance of Na–S battery with Azo–TbTh‐modified separators and Li–SeS_2_ battery with TPB–DMTP‐COF‐modified separators. a) Schematic diagram of the synthesis and application of Azo–TbTh separator of Na–S battery. b) Cycling test of Na–S battery with Azo–TbTh and glass‐fiber separator up to 1000 cycles at 1C. a,b) Reproduced with permission.^[^ ^88^ ^]^ Copyright 2021, American Chemical Society. c) Schematics image of Li–SeS_2_ battery with the TPB–DMTP‐COF separator. d) The long life cycle test of TPB–DMTP‐COF‐modified‐separator‐based Li–SeS_2_ battery at 1C. c,d) Reproduced with permission.^[^ ^92^ ^]^ Copyright 2021, Royal Society of Chemistry.

For instance, Wang and co‐workers have synthesized a kind of COFs‐based thin films with azobenzene side groups (**Figure** **10**a).^[^ ^88^ ^]^ The azobenzene branches possess two advantages: 1) inhibiting the PSs shuttling effect by reducing the pore size to the sub‐nanometer range and 2) accelerating the Na^+^ migration by acting as ion‐hopping sites. Specifically, at 0.2C, the COFs thin film as the separator in Na–S battery shows a reversible capacity of 1295 mA h g^−1^ and a low fading rate of 0.036% per cycle with 1000 cycles at 1C (Figure 10b). This study emphasizes the significance of COFs design for separator modification in improving the Na–S battery performance.

To address the low electronic and ionic conductivity of elemental S reactive substances, elements like Se, Fe, Ti, and Te are doped with S to enhance its electrochemical activity.^[^ ^89^ ^]^ Se is especially appealing among them, and the doping of Se would efficiently facilitate the reaction kinetics of S and inhibit the formation of PSs, where SeS_2_ obtains excellent electronic conductivity and strong ionic conductivity, thus making it to be the best ratio choice.^[^ ^90^, ^91^ ^]^ In addition, SeS_2_ also has a superior capacity of 1342 mAh g^−1^. Nevertheless, the sluggish transportation of Li^+^ and shuttling effect attracted by polysulfides and polyselenides have restricted the application of Li–SeS_2_ battery. For instance, a separator (TPB–DMTP‐COF‐coated separator) reported by Cai et al. can effectively resolve these issues (Figure 10c).^[^ ^92^ ^]^ When TPB–DMTP‐COF applied in separator modification, the accumulation of lithium bis(trifluoromethanesulfonyl)imide (LiTFSI) in channels results in narrower pore size and improved transfer efficiency of Li^+^ in cell. As a consequence, with a SeS_2_ loading of 2 mg cm^−2^, the Li−SeS_2_ battery attains a superior performance with TPB–DMTP‐COF separator of 844.6 mAh g^−1^ at 0.5C. The cells demonstrate a specific capacity of 684 mAh g^−1^ at 1C even with 4 mg cm^−2^ SeS_2_. Moreover, the capacity of the battery can be sustained at 416.3 mAh g^−1^ after 800 cycles (Figure 10d).

In addition, Ke et al. have synthesized a kind of ATFG‐COF fiber (synthesized from 2,4,6‐trihydroxy‐1,3,5‐benzenetrialdehyde and hydrazine) and modified it onto PP that can serve as a coating layer in Li–SeS_2_ battery.^[^ ^93^ ^]^ Experimental results and DFT theoretical calculations show that the ATFG‐COF with abundant carbonyl group could facilitate Li^+^ transport, and trap bis[(trifluoromethyl)sulfonyl]azide anionics (TFSI^−^) through the hydrogen bond generated between TFSI^−^ and the amino group in the channel, thus narrowing the channels and promoting the Li^+^‐transfer number. Hence, ATFG‐COF fiber coating can not only reduce the appearance of Li dendrites by constructing a quick and consistent Li^+^ diffusing layer on the Li anode, but also conquer the shuttling effect by screening polysulfide and polyselenides ions. After 200 cycles at 0.5C, the Li–SeS_2_ cell with the ATFG‐COF/PP separator presents high cycle stability with a capacity of 509 mAh g^−1^.

In addition, some researchers have also designed ionic COFs with anionic or cationic structures that might possess functions of interaction with Li^+^, PSs, or polyselenides. For instance, Zhao et al. have synthesized ATG–DMTZ‐COF (ATG: 1,3,5‐benzenetricarboxaldehyde, DMTZ: 3,5‐diamino‐1,2,4‐triazole) with abundant N sites for Li–SeS_2_ battery separator modification.^[^ ^94^ ^]^ In the pore channels of ATG–DMTZ‐COF, the N atom would preferentially interact with the Li^+^ to create a N–Li bond to promote Li^+^ diffusion during battery cycle processes. Moreover, they demonstrate that the presence of TFSI^−^ in the pore channel will cause the pore size of ATG–DMTZ‐COF to decrease thus inhibiting the shuttling effects of PSs and polyselenides by the sieving effect. Consequently, with a SeS_2_ loading of 2.38 mg cm^−2^, the battery applying the ATG–DMTZ‐COF‐modified separator exhibits exceptional performances with an initial capacity of 1028.7 mAh g^−1^ at 0.5C. Furthermore, after 700 cycles at 1C, a specific capacity of 404.7 mAh g^−1^ can be still maintained.

## 3 Perspective

On account of aforementioned content, we have summarized the applications of COFs‐modified separators in S‐based battery yet there are still some important issues need to be summarized and discussed. As an imperative component in battery, separator plays a profound role in settling problems in S‐based battery and an ideal S‐based battery separator needs to meet the following properties: 1) remarkable electrolyte wettability, high porosity, and high electrochemical stability; 2) excellent mechanical/thermal stability to tolerate dendrite formation; and 3) excellent functions in PSs‐adsorption/catalytic conversion and metal‐ions/electrolyte transport. Currently, its disadvantages such as low wettability, inferior metal‐ions/electrolyte transfer, and shuttling effect of commercial separators hinder the performance of S‐based battery. To address these complicated issues with S‐based battery, modifying commercial separators with functional materials is recognized as one of the most efficient techniques. More importantly, due to the various conditions on both electrode sides, the requirements for the separator are actually diversified. For functional layers facing S cathode, the capacity of PSs adsorption/catalysis should be considered first as well as conductivity for efficient transport of electrons between the electrode and separator. Simultaneously, an anodic interlayer should maintain rapid Li^+^ transfer and possess anode‐protection capability.

The tunable properties of COFs (e.g., high porosity, well‐defined structure, modifiability, and crystallinity) provide much possibility to be applied for S‐based battery separators to meet the requirements of electrode. In terms of COFs material design, previous research has guided the way: 1) homogeneous pore channels facilitate metal‐ion/electrolyte transport; 2) the precise design of the structure allows the introduction of additional and ordered active sites to improve the cell performance; 3) modulation of the COFs nanomorphology (e.g., exfoliated nanosheets, nano‐fiber, etc.) may expose more active sites to improve cell performance; and 4) design of the anionic/cationic backbone to enhance the interactions with the metal ions, PSs, or polyselenides. In terms of the strategy for the preparation of COFs‐modified separators, vacuum filtration, and casting are relatively simple methods that are suitable for most of materials, and allow facile adjustment of the modified layer (e.g., thickness and composition, etc.). However, both methods might still lead to high interfacial resistance and poor interfacial contact. In addition to this, there has been some pioneering method on in situ interfacial polymerization, yet it still faces obstacles like large‐scale production. Thus, it is desirable to explore more powerful synthesis methods to conquer the technical barriers in the production of COFs‐modified separators.

Actually, the exploration of S‐based batteries for potential applications is still at early stage and the achieved performances are still far from satisfactory. For most of reported S‐based battery, they are difficult to achieve high energy density, long cycle stability, and high‐rate performance at the same time. There remains a long way to go to promote the S‐based battery from laboratory investigation to practical application. As the most critical issues of S‐based batteries, the PSs shuttle effect and lithium dendrite problems are most important challenges to overcome. Despite some advanced techniques like solid electrolytes can partially address the PSs shuttle problems, tremendous efforts are still needed to conquer the technical barriers for their potential industrial applications. Moreover, other important issues like the safety, cost, and feasibility in processing also needed to be carefully and systematically studied.

COFs hold much promise to be applied in the separator of S‐based battery to conquer the possible challenges of S‐based battery to some extent and the research directions concentrated on COFs‐modified separators of S‐based battery are summarized as follows: 1) the investigation of COFs‐modified separators mainly focuses on Li–S, Na–S, or Li–SeS_2_ battery systems at current stage and there is a huge demand for the development of more advanced COFs‐modified separators or novel battery systems to achieve better performance; 2) processing methods with enhanced interfacial interaction and low interfacial impedance that can satisfy the demand of industrial production need to be developed; 3) for the functionality of the separator, it is essential to systematically take the requirements for the both sides of cathodic and anodic electrodes into consideration, and directionally design the multifunctional COFs for further applications; and 4) as a kind of crystalline materials, COFs can serve as suitable platforms to study the internal battery mechanisms yet more deeper insights into the structure–property relationships are needed to guide the material design. In this regard, a large number of pioneering works relating to both the design of COFs or investigation of advanced battery techniques would still be essential to further accelerate the potential applications of COFs in the separators of S‐based battery.

## 4 Conclusions

This review first comments the development history and structural characteristics of COFs for S‐based battery separators; then, it summarizes the preparation approaches of COFs‐modified separators containing vacuum filtration, casting, and interfacial in situ polymerization; after that, it gives a systematic review on the design and performance of S‐based battery systems (including Li–S, Na–S, and Li–SeS_2_ battery) based on the requirements of anodic or cathodic electrodes, and finally a perspective and possible research directions in this field are also discussed. Although the applications of COFs in separator modification for S‐based battery have attracted sustained attention, there is a giant gap between the laboratory investigation and industrial applications at current stage. As we envisioned, huge efforts relating to COFs design or separator processing techniques are still required to achieve “lab to industry” technology advance in the next decades. We anticipate this review will offer fresh perspectives or guidance for readers to have a profound understanding of this field and promote the development of novel COFs for S‐based battery applications to expand the limitation of current battery technology.

## Conflict of Interest

The authors declare no conflict of interest.

## References

[1] R. Fang , J. Xu , D.-W. Wang , Energy Environ. Sci. 2020, 13, 432.

[2] C. Ye , D. Chao , J. Shan , H. Li , K. Davey , S.-Z. Qiao , Matter 2020, 2, 323.

[3] Y. Yang , G. Zheng , Y. Cui , Chem. Soc. Rev. 2013, 42, 3018. 23325336 10.1039/c2cs35256g

[4] X. Hong , J. Mei , L. Wen , Y. Tong , A. J. Vasileff , L. Wang , J. Liang , Z. Sun , S. X. Dou , Adv. Mater. 2019, 31, e1802822. 30480839 10.1002/adma.201802822

[5] H. Ye , Y. Li , InfoMat 2022, 4, e12291.

[6] J.-H. Wang , S. Li , Y. Chen , L.-Z. Dong , M. Liu , J.-W. Shi , S.-L. Li , Y.-Q. Lan , Adv. Funct. Mater. 2022, 32, 2210259.

[7] S. Li , J.-H. Wang , L.-Z. Dong , Y. Zhang , X.-M. Yao , Y. Chen , S.-L. Li , Y.-Q. Lan , Chin. Chem. Lett. 2022, 34, 107633.

[8] J.-H. Wang , Y. Zhang , M. Liu , G.-K. Gao , W. Ji , C. Jiang , X. Huang , Y. Chen , S.-L. Li , Y.-Q. Lan , Cell Rep. Phys. Sci. 2021, 2, 100583.

[9] C. Jiang , Y. Zhang , M. Zhang , N.-N. Ma , G.-K. Gao , J.-H. Wang , M.-M. Zhang , Y. Chen , S.-L. Li , Y.-Q. Lan , Cell Rep. Phys. Sci. 2021, 2, 100392.

[10] X. Yu , A. Manthiram , Adv. Funct. Mater. 2020, 30, 2004084.

[11] F. Shi , J. Yu , C. Chen , S. P. Lau , W. Lv , Z.-L. Xu , J. Mater. Chem. A 2022, 10, 19412.

[12] S. H. Chung , A. Manthiram , Adv. Mater. 2019, 31, e1901125. 31081272 10.1002/adma.201901125

[13] Y.-X. Yin , S. Xin , Y.-G. Guo , L.-J. Wan , Angew. Chem., Int. Ed. 2013, 52, 13186. 10.1002/anie.201304762 24243546

[14] R. Fang , S. Zhao , Z. Sun , W. Wang , H.-M. Cheng , F. Li , Adv. Mater. 2017, 29, 1606823. 10.1002/adma.201606823 28380284

[15] Y.-X. Wang , B. Zhang , W. Lai , Y. Xu , S.-L. Chou , H.-K. Liu , S.-X. Dou , Adv. Energy Mater. 2017, 7, 1602829.

[16] Z. W. Seh , Y. Sun , Q. Zhang , Y. Cui , Chem. Soc. Rev. 2016, 45, 5605. 27460222 10.1039/c5cs00410a

[17] G.-K. Gao , Y.-R. Wang , S.-B. Wang , R.-X. Yang , Y. Chen , Y. Zhang , C. Jiang , M.-J. Wei , H. Ma , Y.-Q. Lan , Angew. Chem., Int. Ed. 2021, 60, 10147. 10.1002/anie.202016608 33511739

[18] G.-K. Gao , Y.-R. Wang , H.-J. Zhu , Y. Chen , R.-X. Yang , C. Jiang , H. Ma , Y.-Q. Lan , Adv. Sci. 2020, 7, 2002190. 10.1002/advs.202002190 PMC7740102 33344128

[19] C. Guo , M. Liu , G.-K. Gao , X. Tian , J. Zhou , L.-Z. Dong , Q. Li , Y. Chen , S.-L. Li , Y.-Q. Lan , Angew. Chem., Int. Ed. 2022, 61, e202113315. 10.1002/anie.202113315 34716649

[20] X. Yao , C. Guo , C. Song , M. Lu , Y. Zhang , J. Zhou , H.-M. Ding , Y. Chen , S.-L. Li , Y.-Q. Lan , Adv. Mater. 2023, 35, 2208846. 10.1002/adma.202208846 36444853

[21] J. Wang , L. Si , Q. Wei , X. Hong , L. Lin , X. Li , J. Chen , P. Wen , Y. Cai , J. Energy Chem. 2019, 28, 54.

[22] X. Chen , Y. Wang , J. Wang , J. Liu , S. Sun , L. Zhu , Q. Ma , N. Zhu , X. Wang , J. Chen , W. Yan , J. Mater. Chem. A 2022, 10, 1359.

[23] N. Deng , W. Kang , Y. Liu , J. Ju , D. Wu , L. Li , B. S. Hassan , B. Cheng , J. Power Sources 2016, 331, 132.

[24] W. Luo , S. Cheng , M. Wu , X. Zhang , D. Yang , X. Rui , J. Power Sources 2021, 509, 230372.

[25] L. Fan , M. Li , X. Li , W. Xiao , Z. Chen , J. Lu , Joule 2019, 3, 361.

[26] S. S. Zhang , J. Power Sources 2007, 164, 351.

[27] H. Lee , M. Yanilmaz , O. Toprakci , K. Fu , X. Zhang , Energy Environ. Sci. 2014, 7, 3857.

[28] M. Agostini , J. Hassoun , Sci. Rep. 2015, 5, 7591. 25558001 10.1038/srep07591 PMC5154567

[29] D. E. Fenton , J. M. Parker , P. V. Wright , Polymer 1973, 14, 589.

[30] C. M. Costa , M. M. Silva , S. Lanceros-Méndez , RSC Adv. 2013, 3, 11404.

[31] D. Djian , F. Alloin , S. Martinet , H. Lignier , J. Power Sources 2009, 187, 575.

[32] J. Xu , W. Tang , C. Yang , I. Manke , N. Chen , F. Lai , T. Xu , S. An , H. Liu , Z. Zhang , Y. Cao , N. Wang , S. Zhao , D. Niu , R. Chen , ACS Energy Lett. 2021, 6, 3053.

[33] B. Jung , J. Membr. Sci. 2004, 229, 129.

[34] F. Croce , F. S. Fiory , L. Persi , B. Scrosati , Electrochem. Solid-State Lett. 2001, 4, A121.

[35] Y. Liang , S. Cheng , J. Zhao , C. Zhang , S. Sun , N. Zhou , Y. Qiu , X. Zhang , J. Power Sources 2013, 240, 204.

[36] A. Hashimoto , K. Yagi , H. Mantoku , U.S. Patent, 6,048,607, 2000.

[37] S. Nagou , S. Nakamura , U.S. Patent, 4,791,144, 1988.

[38] T. Yu , U.S. Patent, 6,080,507, 2000.

[39] J. He , Y. Chen , A. Manthiram , Energy Environ. Sci. 2018, 11, 2560.

[40] Z. A. Ghazi , X. He , A. M. Khattak , N. A. Khan , B. Liang , A. Iqbal , J. Wang , H. Sin , L. Li , Z. Tang , Adv. Mater. 2017, 29, 1606817. 10.1002/adma.201606817 28318064

[41] W. Ren , W. Ma , S. Zhang , B. Tang , Energy Storage Mater. 2019, 23, 707.

[42] S.-Y. Ding , W. Wang , Chem. Soc. Rev. 2013, 42, 548. 23060270 10.1039/c2cs35072f

[43] J.-N. Chang , Q. Li , J.-W. Shi , M. Zhang , L. Zhang , S. Li , Y. Chen , S.-L. Li , Y.-Q. Lan , Angew. Chem., Int. Ed. 2023, 62, e202218868. 10.1002/anie.202218868 36581593

[44] J.-N. Chang , Q. Li , Y. Yan , J.-W. Shi , J. Zhou , M. Lu , M. Zhang , H.-M. Ding , Y. Chen , S.-L. Li , Y.-Q. Lan , Angew. Chem., Int. Ed. 2022, 61, e202209289. 10.1002/anie.202209289 35851736

[45] Y.-R. Wang , H.-M. Ding , S.-N. Sun , J.-W. Shi , Y.-L. Yang , Q. Li , Y. Chen , S.-L. Li , Y.-Q. Lan , Angew. Chem., Int. Ed. 2022, 61, e202212162. 10.1002/anie.202212162 36229417

[46] Y.-R. Wang , H.-M. Ding , X.-Y. Ma , M. Liu , Y.-L. Yang , Y. Chen , S.-L. Li , Y.-Q. Lan , Angew. Chem., Int. Ed. 2022, 61, e202114648. 10.1002/anie.202114648 34806265

[47] Z. Wang , S. Zhang , Y. Chen , Z. Zhang , S. Ma , Chem. Soc. Rev. 2020, 49, 708. 31993598 10.1039/c9cs00827f

[48] X. Liu , D. Huang , C. Lai , G. Zeng , L. Qin , H. Wang , H. Yi , B. Li , S. Liu , M. Zhang , R. Deng , Y. Fu , L. Li , W. Xue , S. Chen , Chem. Soc. Rev. 2019, 48, 5266. 31468053 10.1039/c9cs00299e

[49] M. C. Scicluna , L. Vella-Zarb , ACS Appl. Nano Mater. 2020, 3, 3097.

[50] K. Geng , T. He , R. Liu , S. Dalapati , K. T. Tan , Z. Li , S. Tao , Y. Gong , Q. Jiang , D. Jiang , Chem. Rev. 2020, 120, 8814. 31967791 10.1021/acs.chemrev.9b00550

[51] C. Guo , J. Zhou , Y. Chen , H. Zhuang , J. Li , J. Huang , Y. Zhang , Y. Chen , S.-L. Li , Y.-Q. Lan , Angew. Chem., Int. Ed. 2023, 62, e202300125. 10.1002/anie.202300125 36661867

[52] C. Guo , J. Zhou , Y. Chen , H. Zhuang , Q. Li , J. Li , X. Tian , Y. Zhang , X. Yao , Y. Chen , S.-L. Li , Y.-Q. Lan , Angew. Chem., Int. Ed. 2022, 61, e202210871. 10.1002/anie.202210871 35938536

[53] B. Hu , J. Xu , Z. Fan , C. Xu , S. Han , J. Zhang , L. Ma , B. Ding , Z. Zhuang , Q. Kang , X. Zhang , Adv. Energy Mater. 2023, 13, 2203540.

[54] N. Deng , Y. Liu , W. Yu , J. Kang , Q. Li , H. Gao , L. Zhang , W. Kang , Y. Liu , B. Cheng , Energy Storage Mater. 2022, 46, 29.

[55] A. P. Cote , A. I. Benin , N. W. Ockwig , M. O’Keeffe , A. J. Matzger , O. M. Yaghi , Science 2005, 310, 1166. 16293756 10.1126/science.1120411

[56] Y. An , S. Tan , Y. Liu , K. Zhu , L. Hu , Y. Rong , Q. An , Energy Storage Mater. 2021, 41, 354.

[57] J. Yoo , S.-J. Cho , G. Y. Jung , S. H. Kim , K.-H. Choi , J.-H. Kim , C. K. Lee , S. K. Kwak , S.-Y. Lee , Nano Lett. 2016, 16, 3292. 27104986 10.1021/acs.nanolett.6b00870

[58] Q. Xu , K. Zhang , J. Qian , Y. Guo , X. Song , H. Pan , D. Wang , X. Li , ACS Appl. Energy Mater. 2019, 2, 5793.

[59] J. Xu , S. An , X. Song , Y. Cao , N. Wang , X. Qiu , Y. Zhang , J. Chen , X. Duan , J. Huang , W. Li , Y. Wang , Adv. Mater. 2021, 33, 2105178. 10.1002/adma.202105178 34622528

[60] J. Shi , M. Su , H. Li , D. Lai , F. Gao , Q. Lu , ACS Appl. Mater. Interfaces 2022, 14, 42018. 36097371 10.1021/acsami.2c10917

[61] J. Zhao , G. Yan , X. Zhang , Y. Feng , N. Li , J. Shi , X. Qu , Chem. Eng. J. 2022, 442, 136352.

[62] K. Sun , C. Wang , Y. Dong , P. Guo , P. Cheng , Y. Fu , D. Liu , D. He , S. Das , Y. Negishi , ACS Appl. Mater. Interfaces 2022, 14, 4079. 35005891 10.1021/acsami.1c20398

[63] Y. Zhang , C. Guo , J. Zhou , X. Yao , J. Li , H. Zhuang , Y. Chen , Y. Chen , S. L. Li , Y. Q. Lan , Small 2023, 19, e2206616. 36440668 10.1002/smll.202206616

[64] Y. Wang , X. Yang , P. Li , F. Cui , R. Wang , X. Li , Macromol. Rapid Commun. 2022, 2200760. 10.1002/marc.202200760 36385727

[65] J. Wang , L. Si , Q. Wei , X. Hong , S. Cai , Y. Cai , ACS Appl. Nano Mater. 2017, 1, 132.

[66] J.-L. Qin , B.-Q. Li , J.-Q. Huang , L. Kong , X. Chen , H.-J. Peng , J. Xie , R. Liu , Q. Zhang , Mater. Chem. Front. 2019, 3, 615.

[67] B.-Q. Li , H.-J. Peng , X. Chen , S.-Y. Zhang , J. Xie , C.-X. Zhao , Q. Zhang , CCS Chem. 2019, 1, 128.

[68] Q. X. Shi , H. J. Pei , N. You , J. Wu , X. Xiang , Q. Xia , X. L. Xie , S. B. Jin , Y. S. Ye , Chem. Eng. J. 2019, 375, 121977.

[69] B. Hu , B. Ding , C. Xu , Z. Fan , D. Luo , P. Li , H. Dou , X. Zhang , Nanomaterials 2022, 12, 255. 35055272 10.3390/nano12020255 PMC8779782

[70] R. Wang , Q. Cai , Y. Zhu , Z. Mi , W. Weng , Y. Liu , J. Wan , J. Hu , C. Wang , D. Yang , J. Guo , Chem. Mater. 2021, 33, 3566.

[71] Y. Cao , H. Wu , G. Li , C. Liu , L. Cao , Y. Zhang , W. Bao , H. Wang , Y. Yao , S. Liu , F. Pan , Z. Jiang , J. Sun , Nano Lett. 2021, 21, 2997. 33764070 10.1021/acs.nanolett.1c00163

[72] X. Deng , Y. Li , L. Li , S. Qiao , D. Lei , X. Shi , F. Zhang , Nanotechnology 2021, 32, 275708. 10.1088/1361-6528/abf211 33765671

[73] K. Zhang , X. Li , L. Ma , F. Chen , Z. Chen , Y. Yuan , Y. Zhao , J. Yang , J. Liu , K. Xie , K. P. Loh , ACS Nano 2023, 17, 2901. 36638084 10.1021/acsnano.2c11300

[74] Y. Zhu , J. Yang , X. Qiu , M. Li , G. He , Q. Wang , Z. Xie , X. Li , H. Yu , ACS Appl. Mater. Interfaces 2021, 13, 60373. 34902968 10.1021/acsami.1c19705

[75] J. Wang , W. Qin , X. Zhu , Y. Teng , Energy 2020, 199, 117372.

[76] W. Yan , X. Gao , J.-L. Yang , X. Xiong , S. Xia , W. Huang , Y. Chen , L. Fu , Y. Zhu , Y. Wu , Small 2022, 18, 2106679. 10.1002/smll.202106679 35060309

[77] M. Li , G. Yan , P. Zou , H. Ji , H. Wang , Z. Hu , Z. Yang , Y. Feng , H. Ben , X. Zhang , ACS Sustainable Chem. Eng. 2022, 10, 13638.

[78] C. Jiang , M. Tang , S. Zhu , J. Zhang , Y. Wu , Y. Chen , C. Xia , C. Wang , W. Hu , Angew. Chem., Int. Ed. 2018, 57, 16072. 10.1002/anie.201809907 30295985

[79] Y. Cao , C. Liu , M. Wang , H. Yang , S. Liu , H. Wang , Z. Yang , F. Pan , Z. Jiang , J. Sun , Energy Storage Mater. 2020, 29, 207.

[80] P. Li , H. Lv , Z. Li , X. Meng , Z. Lin , R. Wang , X. Li , Adv. Mater. 2021, 33, 2007803. 10.1002/adma.202007803 33734507

[81] L. Han , S. Sun , Y. Yang , J. Yue , J. Li , Appl. Surf. Sci. 2023, 610, 155496.

[82] G.-H. Li , Y. Yang , J.-C. Cai , T. Wen , L.-C. Zhuang , X.-Y. Huang , Y.-P. Cai , X.-J. Hong , ACS Appl. Energy Mater. 2022, 5, 13554.

[83] Z. Li , W. Ji , T. X. Wang , Y. Zhang , Z. Li , X. Ding , B. H. Han , W. Feng , ACS Appl. Mater. Interfaces 2021, 13, 22586. 33951910 10.1021/acsami.1c04517

[84] T. Li , J. Xu , C. Wang , W. Wu , D. Su , G. Wang , J. Alloys Compd. 2019, 792, 797.

[85] K. B. Hueso , M. Armand , T. Rojo , Energy Environ. Sci. 2013, 6, 734.

[86] L. Wang , T. Wang , L. Peng , Y. Wang , M. Zhang , J. Zhou , M. Chen , J. Cao , H. Fei , X. Duan , J. Zhu , X. Duan , Natl. Sci. Rev. 2022, 9, nwab050. 35401989 10.1093/nsr/nwab050 PMC8986459

[87] Q. Guo , Z. Zheng , Adv. Funct. Mater. 2020, 30, 1907931.

[88] C. Yin , Z. Li , D. Zhao , J. Yang , Y. Zhang , Y. Du , Y. Wang , ACS Nano 2022, 16, 14178. 35994525 10.1021/acsnano.2c04273

[89] S. Li , W. Zhang , Z. Zeng , S. Cheng , J. Xie , Electrochem. Energy Rev. 2020, 3, 613.

[90] J. Hu , Y. Ren , L. Zhang , J. Power Sources 2020, 455, 227955.

[91] A. Abouimrane , D. Dambournet , K. W. Chapman , P. J. Chupas , W. Weng , K. Amine , J. Am. Chem. Soc. 2012, 134, 4505. 22364225 10.1021/ja211766q

[92] Y. Yang , X.-J. Hong , C.-L. Song , G.-H. Li , Y.-X. Zheng , D.-D. Zhou , M. Zhang , Y.-P. Cai , H. Wang , J. Mater. Chem. A 2019, 7, 16323.

[93] J. Wang , J.-H. Chen , Z.-C. Chen , Z.-Y. Wu , X.-N. Zhong , J.-P. Ke , Coatings 2022, 12, 289.

[94] J. Wang , J.-P. Ke , Z.-Y. Wu , X.-N. Zhong , S.-B. Zheng , Y.-J. Li , W.-H. Zhao , Coatings 2022, 12, 931.

